# morFeus: a web-based program to detect remotely conserved orthologs using symmetrical best hits and orthology network scoring

**DOI:** 10.1186/1471-2105-15-263

**Published:** 2014-08-06

**Authors:** Ines Wagner, Michael Volkmer, Malvika Sharan, Jose M Villaveces, Felix Oswald, Vineeth Surendranath, Bianca H Habermann

**Affiliations:** Max Planck Institute of Biochemistry, Am Klopferspitz 18, Martinsried, 82152 Germany; IMIB, Julius-Maximilians-University Würzburg, Josef-Schneider-Strasse 2 - Building D15, Würzburg, 97080 Germany; Section Physics of Living Systems, Department of Physics and Astronomy & Laser Centre, VU University Amsterdam, De Boelelaan 1081, Office U0.30, Amsterdam, 1081 HV Netherlands; Max Planck Institute of Molecular Cell Biology and Genetics, Pfotenhauerstrasse 108, Dresden, 01307 Germany

**Keywords:** Remote sequence conservation, Orthology, Alignment clustering, Reciprocal best hit, Orthology network, Eigenvector centrality, Meta-analysis based orthology finder using symmetrical best hits

## Abstract

**Background:**

Searching the orthologs of a given protein or DNA sequence is one of the most important and most commonly used Bioinformatics methods in Biology. Programs like BLAST or the orthology search engine Inparanoid can be used to find orthologs when the similarity between two sequences is sufficiently high. They however fail when the level of conservation is low. The detection of remotely conserved proteins oftentimes involves sophisticated manual intervention that is difficult to automate.

**Results:**

Here, we introduce morFeus, a search program to find remotely conserved orthologs. Based on relaxed sequence similarity searches, morFeus selects sequences based on the similarity of their alignments to the query, tests for orthology by iterative reciprocal BLAST searches and calculates a network score for the resulting network of orthologs that is a measure of orthology independent of the E-value. Detecting remotely conserved orthologs of a protein using morFeus thus requires no manual intervention. We demonstrate the performance of morFeus by comparing it to state-of-the-art orthology resources and methods. We provide an example of remotely conserved orthologs, which were experimentally shown to be functionally equivalent in the respective organisms and therefore meet the criteria of the orthology-function conjecture.

**Conclusions:**

Based on our results, we conclude that morFeus is a powerful and specific search method for detecting remotely conserved orthologs. morFeus is freely available at http://bio.biochem.mpg.de/morfeus/. Its source code is available from Sourceforge.net (https://sourceforge.net/p/morfeus/).

**Electronic supplementary material:**

The online version of this article (doi:10.1186/1471-2105-15-263) contains supplementary material, which is available to authorized users.

## Background

Trying to find the orthologs of a given protein or DNA sequence has co-evolved with sequencing itself. Fitch defined the terms *orthology* and *paralogy* as early as 1970, when only very few protein sequences were known [1]. With the advent of fully sequenced genomes, the computational study of orthologous protein relationships in evolution, comparative genomics, but also for substantiating the evolutionary conservation of fundamental cellular processes increased exponentially. It is widely accepted and has been proven in many cases that orthologs typically have equivalent functions across organisms [2]. Transferring the functional annotation of a protein to its orthologs in other species is therefore routine in genome annotation. Virtually all genome centres provide information on orthologous protein families ([3–6], and see also [7]).

Two proteins that are each others best hit (also known as reciprocal best hit (RBH) or symmetrical best hit) in a pair-wise genome comparison are considered orthologous. Protein families are in practice more complicated, as genomes have evolved substantially, leading amongst others to gene duplications and losses [2]. Yet, reciprocal sequence similarity is thus far one of the main established methods for defining orthology computationally and is ubiquitously used on a small- as well as a large-scale. Other orthology search methods combine sequence-based searches with phylogenetic methods or graph-clustering algorithms to circumvent computationally intense phylogenetic calculations. These include Berkeley PHOG [8], FAT-CAT [9], TreeFam [10], PhylomeDB [11], EnsembleCompara [12], and OrthoMCL [13].

Due to high sequence divergence, many true orthologs are only discovered using more sophisticated techniques like profile-based database searches (PSI-BLAST [14], HMMER [15, 16]), profile-profile comparisons (HHblits [17], HHsenser [18]) or drastically relaxed E-value thresholds. All those approaches exploit the fact that members of orthologous protein families, even if they are strongly diverged, still share a common sequence pattern. Though powerful in finding more remotely conserved orthologs, profile-based methods are prone to profile drift (see for instance [19, 20], or [21]). Manual comparison of sequence alignments is oftentimes used to detect remotely conserved orthologs in the twilight zone. Virtually all above-mentioned approaches are hard to run in an unsupervised manner. Szklarczyk and colleagues [22] have introduced an iterative orthology prediction pipeline based on reciprocal best-hit assessment, Ortho-Profile, that performs sequence-to-sequence, profile-to-sequence and HMM-to-HMM comparisons in a step-wise process to uncover remotely conserved orthologs. Though very powerful in detecting remotely conserved orthologs, there is to date no ready-to-use script or web-interface of Ortho-Profile available. This makes using Ortho-Profile for non-experts difficult, representing a true drawback of the software.

With morFeus, we introduce the first, web-based approach to assign remotely conserved orthologs in an unsupervised manner. To explore a substantial part of sequence space, morFeus uses BLAST with relaxed E-value thresholds. It exploits the conserved sequence pattern of orthologs by clustering the alignments of hits to the query. *Bona fide* orthologs serve to verify potential orthologs by the RBH-rule in iterative reciprocal BLAST searches. Finally, a score independent of the BLAST E-value, which is based on the network of orthology, is introduced to describe orthologous relationships. We have determined the accuracy and precision of morFeus by testing its performance against a subset of the HomoloGene database [23], as well as Inparanoid [7]. We demonstrate the sensitivity of morFeus using a set of remotely conserved, mitochondrial protein families that were first uncovered using Ortho-Profile, as well as an example of a remotely conserved, orthologous family, whose members were shown to have identical functions in their respective organisms [24]. morFeus is freely available as a web server at http://bio.biochem.mpg.de/morfeus/. We have submitted its source-code (Additional file 1) to Sourceforge.net (https://sourceforge.net/p/morfeus/) and its virtual machine can be requested from the authors.

## morFeus web server implementation

### The morFeus web server

**Figure 1:**
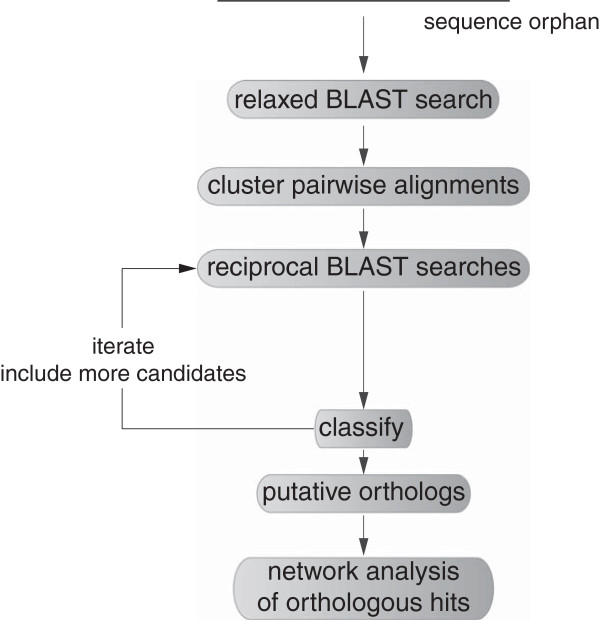
**Workflow of a morFeus search.** morFeus starts with a BLAST search using relaxed E-value settings, clusters all resulting alignments based on their similarity to each other, carries out reciprocal BLAST searches for selected orthology candidates in an iterative manner and after classification of candidates, calculates a network score based on the connectivity of each protein in a network of orthology.

The workflow of the software is shown in Figure 1. A full description of the methods and algorithms used in morFeus can be found in Additional file 2.

#### Relaxed BLAST

A morFeus search starts with a BLAST search (blast+, version 2.2.27) [14], against a protein sequence database using relaxed parameters (default E-value threshold: 100). We recommend an E-value cut-off of at least 100 for sequences without any apparent homolog in distant species, as it covers a reasonably large sequence space. For sequences with clear homologs in distant species, the E-value can be reduced (E-value < = 10). Currently, the user can choose to search against the entire RefSeq protein database of the NCBI or subsets thereof (Bacteria, Eukaryota, Opisthokonta, plants). The choice of E-value cut-off and database will influence the run-time of morFeus (high E-value thresholds and large search space increase the run-time).

#### Distance-based clustering of alignments

All pair-wise alignments of the query BLAST search are clustered based on their similarity to each other. Each alignment is transformed into binary format representing the matches (1) and mismatches (0) of the query with the subject. To strengthen the contribution of rare amino acids, we use the weights of the OPTIMA substitution matrix [25] for the amino acid sequence of the query to calculate the similarity score  of two alignments  and  treating identical and conserved positions as equal.  is further used for distance-based hierarchical clustering with a modified average linkage approach. The conserved positions between a new alignment and the alignments in a cluster are not considered by the classical average linkage approach, as it only calculates the distance between  of the new alignment and the average of all  of an established cluster. We therefore calculate the distance score based on the conserved consensus of the alignments within one cluster and a new alignment (or another cluster).

#### Cluster cutting

The resulting hierarchical tree is analysed with respect to its structure for subsequent cluster splitting. In brief, each hierarchical tree is cut based on its distribution of distances. Based on the analysis of 254, randomly chosen protein families, we determined that an exponential function is the best-suited mathematical model to describe the majority of datasets (97% of tested families; see Additional file 2: Figure S1 and Additional file 3: Table S1). The climbing rate of the exponential function is used to identify cluster boundaries and to cut the tree into individual clusters. A small climbing rate describes highly similar alignments; the steeper the climbing rate, the more dissimilar the alignments will be. We therefore cut the tree at the position where the climbing rate accelerates from a flat to a steep curve. At this point, two more distantly related clusters are linked. A detailed description of the clustering approach, as well as the definition of clusters of the distance tree can be found in Additional file 2.

#### Iterative reciprocal BLAST

Each orthology candidate is submitted to a reciprocal BLAST search and evaluated for its fitness to become a *bona fide* ortholog. In order to maximize the benefit from the RBH hypothesis, several additional features have been implemented in morFeus’ reciprocal BLAST searches: 1) morFeus does several cycles of reciprocal BLASTs, taking the output of the previous rounds into account for selecting orthology candidates and deciding on orthology relationships; hence, morFeus considers not only the query but also all *bona fide* orthologs when deciding on the orthology of novel candidates; 2) if a protein is selected for reciprocal BLAST, morFeus includes all proteins present in its respective candidate cluster for reciprocal BLAST searches; 3) all sequences that are found as RBH by more than two verified orthologs are likewise selected for reciprocal BLASTs. To start iterative reciprocal BLASTs, all sequences with more than 80% identity to the query are selected, as are all sequences that are located within the query cluster. In the first round, only the query is taken to decide on the orthologous relationship of a candidate. For all candidates with an E-value < 10–5, we strictly apply the RBH-rule. However, for sequence relationships with a statistically less reliable E-value (>10-5), it cannot be excluded that the second or even the third hit in a species is the true ortholog [2]. An orthology candidate is only excluded from further analysis when it is rejected by more than 33% of *bona fide* orthologs as a RBH. Reciprocal BLAST searches stop once no new orthology candidates are found.

#### Orthology network construction and centrality scoring

Once relationships between orthologs based on reciprocal BLASTs have been established, morFeus constructs a network of orthology, which reflects the binary relationships between the included sequences. In the orthology network, we discriminate between best-best (bb), best-acceptable (ba), acceptable-acceptable (aa) relationships, as well as one-sided relationships of the type best (b) and acceptable (a). The latter reflect situations, where only one of the two proteins finds the other by BLAST. The type of relationships (edges) between the proteins (nodes) enables us to score the individual candidates using centrality scoring. More precisely, we apply Eigenvector centrality [26] as implemented in NetworkX [27] to score each individual node in the orthology network. To assign initial scores, we use the type of connection between the nodes with descending values: bb = 1, ba = 0.5, aa = 0.25, b = 0.125, a = 0.0625. We use the centrality score as the network score for each node, as it represents a measure of similarity of a node to the group of collected orthologs that is independent of the BLAST E-value.

## Results

### morFeus output

#### Description of web output

**Figure 2:**
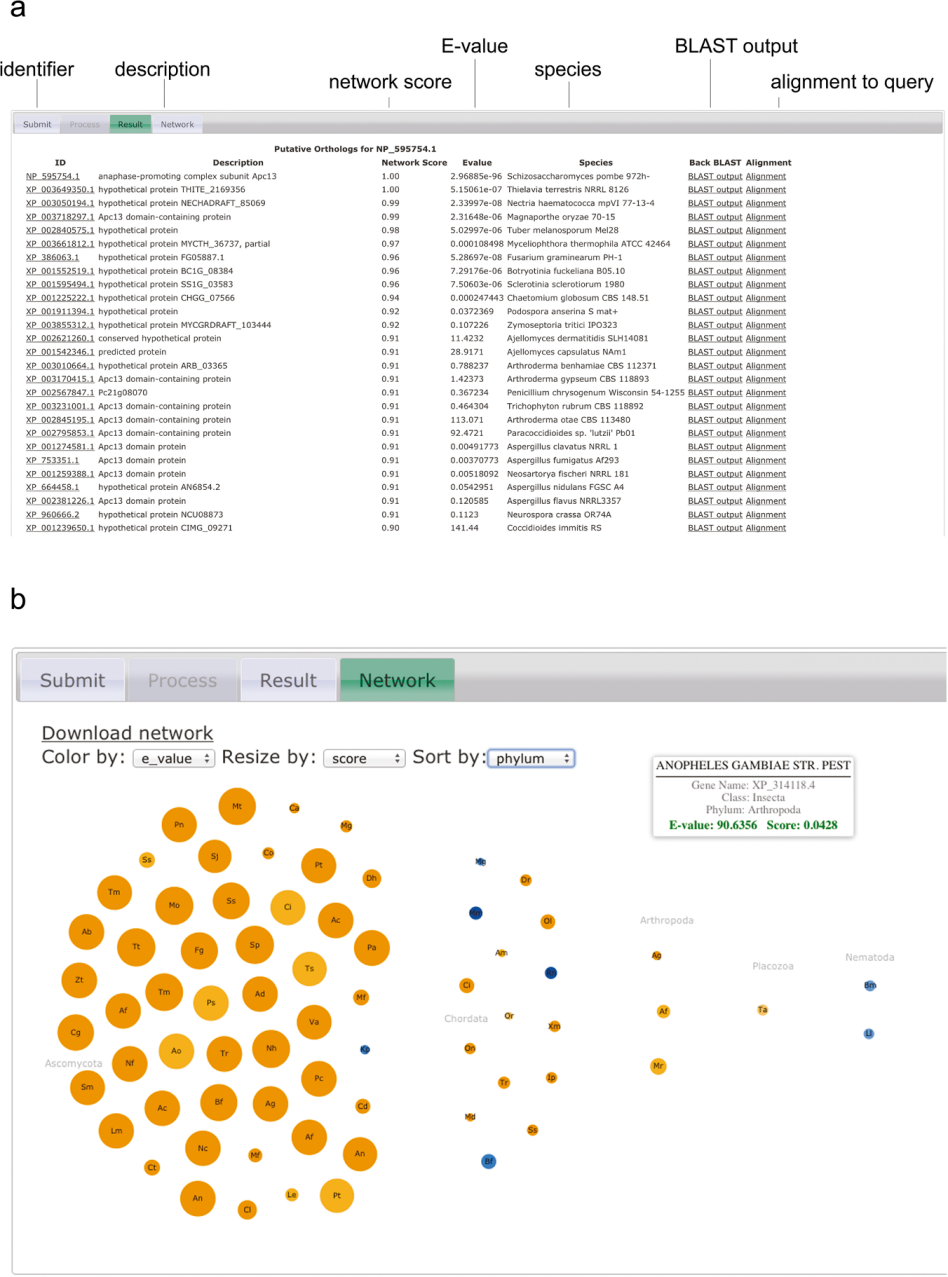
**Output of a morFeus search. (a)** The first couple of hits in the *results* section of a morFeus search. Identified orthologs of the input query (in this case *S. pombe* Apc13) are displayed on the web-site. Parameters describing the hits include the Network Score, as well as the E-value. The BLAST-output of the reciprocal BLAST search, as well as the alignment of the hit to the query are linked from the hit-list. The full list is shown in Additional file 2: Figure S2. **(b)** The network of the hits is displayed on the *network* link of the morFeus output pages. Nodes are coloured by E-value (small E-values = orange, large E-values = blue) and the size of the nodes corresponds to their network score. In the figure shown, the network has been sorted according to phylum. Mouse-over of the nodes displays the species name, the RefSeq ID, Class and Phylum, as well as the E-value and network score of the node as exemplified by the hit from *Anopheles gambiae.*

**Figure 3:**
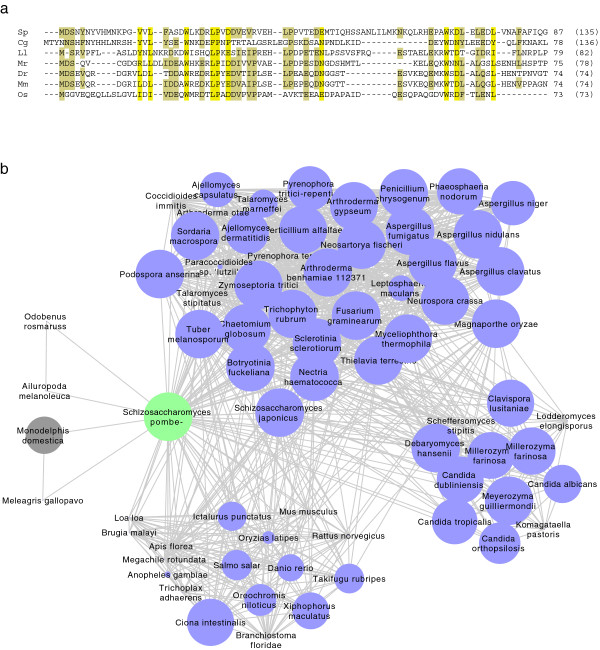
**Alignment and network of orthology of the Apc13 family. (a)** Multiple sequence alignment of some Apc13 orthologs, including those from *Candida glabrata* and *Oryza sativa*. Conserved positions across all shown species are highlighted in bright yellow, those that are conserved in five out of the seven sequences are highlighted in dark yellow. Species abbreviations and accession numbers are listed in Additional file 2: Table S14. **(b)** The network of orthology for the Apc13 family displayed in Cytoscape. There are three tightly connected clusters representing the metazoan and two fungal groups. The false positive predictions are clearly separated from the interconnected clusters (grey nodes). Nodes are scaled according to E-value with low E-values having large circles and high E-values having small ones. An edge-weighted spring-embedded layout was chosen.

The output of a morFeus search is a list of putative orthologs, which have passed the orthology test of the morFeus pipeline (see Additional file 2: Table S2 and Additional file 3: Table S3 for identified orthologs of *Schizosaccharomyces pombe* (*S. pombe, Sp*) Apc13 (NP_595754), and Figure 2 and Additional file 2: Figure S2 a for its web-based output). Next to the NCBI identifier and the species of the hit, its network score and E-value are shown. The user can retrieve the original BLAST search of each hit, as well as the individual BLAST alignments of identified orthologs to the query. The network of orthology is displayed using d3.js (http://d3js.org and see Figure 2b) and can be downloaded as a network file (.sif-format) along with an attribute file that includes information on E-value, score and species for each hit. Both can be imported into Cytoscape for further analysis ([28], see Figure 3b).

#### MorFeus results for the protein family Apc13

As an example of a highly diverged protein family, we chose *S. pombe* Apc13 as a query, a subunit of the Anaphase Promoting Complex that is remotely conserved from yeast to man [24]. There is no HomoloGene group assigned to fission yeast Apc13. The ANAPC13 HomoloGene group from eukaryotes only includes vertebrates. Likewise, Inparanoid failed to detect any orthologs in metazoans for this fission yeast protein. Of the phylogeny software mentioned above, none could complete this protein family from fungi to mammals.

morFeus found 700 hits for *Sp* Apc13 with our settings (E-value of 1000, database RefSeq-opisthokonta) and after 380 reciprocal BLAST searches, it identified 70 orthologs from fungi, nematodes, arthropods, vertebrates and mammals (Figure 2 and Additional file 2: Figure S2, Table S2 and Additional file 3: Table S3; see Figure 3 a for a multiple sequence alignment of a subset of Apc13 orthologs). morFeus readily discovered orthologs based on the similarity of their alignments (Additional file 2: Figure S3 a) and was able to discriminate between false positive and true positive hits solely based on a family-specific conservation pattern: although mouse Apc13 is only the 3rd BLAST-hit from *Mus musculus*, morFeus distinguished its sequence as the orthologous one (Additional file 2: Figure S3 b). morFeus is thus able to effectively distinguish true positive orthologs from a large number of hits in relaxed BLAST searches (Additional file 3: Table S3). 70 of the initial 700 hits are identified by morFeus as orthologs. 66 hits in the initial BLAST are true positive Apc13 orthologs. Only one of the orthologs is not found by morFeus: *Strongylocentrotus purpuratus* Apc13-like protein (XP_001182211) is rejected, because a second, nearly identical sequence exists in the RefSeq database (XP_001184631). The two sequences exclude themselves due to the RBH-rule. While morFeus did not find Apc13 orthologs from all species, the identified sequences from different phyla can retrieve most missing family members from their respective phylum with a standard BLAST search. Four of the identified 70 sequences are false positives (Additional file 2: Table S2 and Additional file 3: Table S3, see Additional file 2: Figure S3 c for pair-wise alignments of false positive identifications). This amounts to a Precision of 93% for the remotely conserved Apc13 protein family. Note that Recall, Precision and Accuracy of morFeus will differ for each protein family. Additional file 3: Table S11 lists Precision values for other, remotely conserved protein families found by morFeus. morFeus results currently exclude all hits that are found as a RBH by the query alone. With this setting, we most likely miss some true positives. None of the *Saccharomycetae* orthologs have been found, even though they are known (Swm1 for *Saccharomyces cerevisiae*). Yet, the number of false positives rises when the query alone is sufficient to include a potential orthologous sequence.

*S. japonicus* Apc13 identifies more vertebrate and mammalian Apc13 members than *S. pombe* and also produces no false positive hits (Precision = 100%), when submitted to morFeus (Additional file 2: Table S4). We have observed this in other protein families as well. This is not surprising, as each query will find a slightly different set of hits in a BLAST search. The more divergent two input queries from the same protein family, the more sequence space can consequently be covered. We therefore recommend using more than one member of a protein family as morFeus queries.

### Performance in detecting orthologs of conserved protein families

We tested whether morFeus could find well-conserved orthologs that are annotated in public resources. We therefore submitted a subset of 190 protein families from the HomoloGene database [3], which we hereafter refer to as the HomoloGene test set, to morFeus and Inparanoid. We focused on proteins with no or a maximum of one conserved domain to mimic sequence orphans. We used the sequences from *S. pombe* as queries and searched against the RefSeq protein database (database RefSeq-opisthokonta, E-value cut-off of 10). Results are shown in Table 1, original data can be found in Additional file 3: Tables S5-S9.

**Table 1:** **Performance of morFeus, HomoloGene and Inparanoid**

Comparison	Recall	Precision	Accuracy	F1-score
HomoloGene - morFeus	86%	94%	99%	89%
Inparanoid - morFeus	85%	94%	98%	88%
HomoloGene - Inparanoid	83%	91%	99%	85%
Inparanoid - HomoloGene	66%	90%	98%	73%

morFeus reached a Recall of 86% and a Precision of 94% when compared against the HomoloGene database, resulting in an F1-score of 89%. Due to the high number of BLAST hits – and therefore true negatives, morFeus’ Accuracy amounted to 99%.

Next, we compared morFeus results of the HomoloGene test set against Inparanoid orthology searches. Results were very similar, with 85% Recall, 94% Precision, an F1-score of 88% and an Accuracy of 98%. Finally, we compared the results from HomoloGene and Inparanoid with each other. When we took HomoloGene as a basis, Inparanoid reached a Recall of 83% and a Precision of 91%, giving an F1-score of 85% and an Accuracy of 99% (300 BLAST hits were considered as true negatives). HomoloGene, when compared to Inparanoid only had a Recall of 66%. This is mostly due to the fact that in conflicting protein family situations, HomoloGene does not assign an ortholog, while Inparanoid does. The Precision was comparable to the other test situations with 90%, resulting in an F1-score of 73% and an Accuracy of 98%.

Based on our data we conclude that morFeus is an accurate and efficient method to detect conserved orthologs and is in its overall performance comparable to the HomoloGene resource, as well as the orthology search engine Inparanoid. We could not observe a high number of false positives. morFeus could indeed complete further 16 (or 8% of) families that were annotated only in fungi and/or plants with orthologs from nematodes, arthropods and vertebrates. In total, morFeus found additional 90 orthologs for the HomoloGene test set (see Additional file 3: Table S10).

### Comparison of morFeus with Ortho-Profile: detecting remotely conserved, mitochondrial proteins in higher eukaryotes

Recently the remote orthology search engine Ortho-Profile was published [22] and applied to the set of mitochondrial proteins from budding and fission yeast. The authors could assign a human ortholog to ~600 proteins from *S. cerevisiae* and/or *S. pombe*. Mitochondrial localization in human cells was experimentally verified for 12 of those proteins. We took the 12 Candidate COX assembly factors from *S. cerevisiae* described in [22] and submitted them to morFeus to determine, whether our method is equally successful in finding their human orthologs (Table 2 and Additional file 3: Table S11, E-value cut-off was 100, database RefSeq-opisthokonta). For six of the 12 proteins, morFeus readily found the human (or at least one vertebrate) ortholog with the yeast protein (Cox20, Cox23, Pet117, Pet191, Pet309 and Coa6, respectively). In all cases, morFeus found the same human/vertebrate ortholog as Ortho-Profile, except for Pet309, where it identified the mitochondrial pentatricopeptide repeat-containing protein LRPPRC instead of PTCD1 (mitochondrial pentatricopeptide repeat-containing protein 1) as the ortholog in metazoans. In four of the cases, Coa1, Coa3, Mss51 and Pet100, morFeus identified the human ortholog via an intermediate species. *S. pombe* was chosen for Coa1 and Coa3; *Branchiostoma floridae* was chosen as the chordate hit for Mss51 and *Schizophyllum commune* for Pet100. morFeus faced a challenge with Cox14 and Cox24, as the similarity in both cases is limited to a very short region even between closely related orthologs from Ascomycota. With the *S. cerevisiae* proteins, we did not succeed to find any ortholog outside of Ascomycota and in case of Cox24, we only found the human ortholog that Ortho-Profile predicts, AURKAIP1, when using the ortholog (identified by standard BLAST-searches) from *Schizosaccharomyces japonicus*. We have calculated the Precision for all searches performed for the Candidate COX assembly factors (Table 2, Additional file 3: Table S11). Except for Cox14, for which morFeus failed to detect orthologs in higher organisms, all proteins reached a Precision of close to 100% (the average Precision was 97%). We also searched for predicted orthologous groups of those 12 proteins by other algorithms (see Additional file 2: Table S12). FAT-CAT and Ortho-MCL performed best and both correctly identified the families for four of the COX assembly factors (Cox23, Pet117, Pet191 and Coa6). The COG database [29] contains the mammalian orthologs only for Cox23 and Coa6 and at least discovered the invertebrate orthologs for Pet191. The families of Cox23, Pet191 and Coa6 were also correctly recognized by eggNog [30]. Finally, Berkley PHOG only found fungal orthologs for Cox14, Cox20, Cox23, Mss51 and Coa3. Next to Ortho-Profile, morFeus is thus the only search engine to identify remotely conserved members for most of the COX assembly factors.

**Table 2:** **Identification of remotely conserved, experimentally verified mitochondrial proteins using morFeus**

Gene name yeast	RefSeq ID	Ortho-profile phase	Gene name vertebrate/human	RefSeq ID vertebrate/human	Found with morFeus	Intermediate species	Precision
COX14	NP_013577	HMM	COX14	NP_116290	No		82%
COX20	NP_010517	Profile	FAM36A (*M. mulatta*)	NP_001244714	Yes		99%
COX23	NP_011984	Sequence	CHCHD7	NP_077276	Yes		91%
COX24	NP_013305	HMM	AURKAIP1	NP_060370	No	Only found with *S. japonicus,* finds *S. cerevisiae* Pet20 (NP_015166) as ortholog	100% (98%)
COA1	NP_012109	HMM	COA1	NP_060694	Yes	*S. pombe*	100% (100%)
COA3	NP_076894	HMM	COA3 homolog	NP_001035521	Yes	*S. pombe*	97% (100%)
MSS51	NP_013304	Profile	MSS51 homolog	NP_001019764	Yes	*B. floridae*	99%
PET100	NP_010364	Profile	Pet100 Homolog	XP_005625312	Yes	*S. commune*	91% (100%)
PET117	NP_010979	Sequence	PET117 homolog	NP_001158283	Yes		100%
PET191	NP_012568	Sequence	COA5	NP_001008216	Yes		100%
PET309	NP_013168	Profile	LRPPRC	NP_573566	Yes		100%
YMR244C-A (COA6)	NP_013972	Sequence	COA6	NP_001013003	Yes		100%

Precision values in brackets are those of the intermediate Species.

We next took all 598 proteins that contained assigned human orthologs from [22] to further test the performance of morFeus on large-scale (E-value was 100, database RefSeq-opisthokonta). We eliminated all proteins that already had *bona fide* orthologs in higher eukaryotes assigned by HomoloGene and searched with those 184 proteins that did not contain any orthologs from Opisthokonta (Additional file 3: Table S13). 8 searches were stopped, as more than 1500 hits were found, suggesting a multi-branching family with sufficient sequence similarity for phylogenetic methods. For 150 (86%) of the remaining 176 proteins, morFeus readily discovered the fission yeast (if available), as well as vertebrate/mammalian ortholog. In 21 cases (12%), an identified ortholog from the morFeus search with the budding yeast protein was used to retrieve orthologs in higher eukaryotes in a subsequent morFeus run. The use of intermediate species is one of the recommended procedures to discover very distantly related orthologs in other species. Five of the 176 proteins were members of multi-branching families with at least one gene duplication in *S. cerevisiae*. In all those cases, the yeast paralog was the putative sequence ortholog assigned by Ortho-Profile. It is for this reason that no ortholog was detected using morFeus. Taken together, we conclude that morFeus is as efficient as Ortho-Profile in discovering remotely conserved orthologs with the advantage of a ready-to-use web interface.

## Discussion

morFeus is a new, web-based method to assign remotely conserved orthologs. Based on sampling of a large part of the sequence space due to relaxed E-value settings, the comparison of pair-wise sequence alignments and iteratively establishing reciprocal similarity relationships, our software is able to efficiently identify orthologs with high sequence divergence. We introduce a measure of orthology independent of the E-value, which is based on the connectivity of sequences in a network of orthology. morFeus searches a large part of sequence space and can detect more divergent family members. This is demonstrated with the help of the remotely conserved, mitochondrial protein families introduced by [22], as well as the example we chose (Apc13 from *S. pombe*). morFeus is so far the first web-based, ready-to-use software that can reliably detect remotely conserved orthologs of a protein in an unsupervised manner.

Ortho-Profile is in our view the most similar search engine to morFeus. It is designed to detect remotely conserved orthologs by a step-wise procedure to identify them based on the similarity of either their sequences, their sequence profiles or their HMMs. Unlike morFeus, Ortho-Profile does not have a ready-to-use web-interface. It is therefore difficult to use for non-experts, which is one of the main target groups for morFeus. As Ortho-Profile partly relies on sequence profiles and HMMs, respectively, it is also not clear, how specific the pipeline is in multi-branching – and also multi-domain families.

Though we consider morFeus very powerful in finding remote orthologs, we acknowledge its limitations: First, morFeus relies fully on BLAST results. If an orthologous sequence is not present in the sampled sequence space or if BLAST fails to detect the sequence with the chosen settings, morFeus will not list it as an ortholog, as is the case in the Apc13 family. Though the ortholog of *S. cerevisiae* Apc13 is known, *Sp* Apc13 does not find it in its initial BLAST search; thus, morFeus fails equally to report this sequence as an Apc13 ortholog. This limitation may be overcome in many cases by using PSI-BLAST instead of BLAST for the initial sequence search, a feature we are planning to implement in future releases of morFeus. We furthermore observed that the success of a morFeus search depends partly on the chosen query sequence. We generally recommend using more than one of the *bona fide* orthologs as a query for a morFeus search to detect more and also more divergent members of an orthologous family. Second, the Eigenvector centrality scores that are calculated for nodes are not discriminative at low values. This is not unexpected as true positives have in some cases a best-best (or best-acceptable) relationship to only two or a few members of an orthologous family. It is for this reason that we do not exclude putative orthologs based on a low network score. morFeus’ network score is however discriminative at large values and can be used as an independent measure to ascertain an orthologous relationship. Third, morFeus might not be able to distinguish between orthologs and paralogs in all cases. This is a result of our procedure to include or exclude orthology candidates based on their relationship to *bona fide* orthologs. We only exclude candidates that are rejected by more than 33% of *bona fide* orthologs as a RBH. By raising this exclusion cut-off, we lose many true positive hits. For the intended use cases of morFeus, where virtually no ortholog is found in more divergent species, finding two potential co-orthologs is better than finding none. Further analysis of the identified sequences using for instance phylogenetic analysis can bring final clarity to the sequence relationships. One possibility to overcome this in our software would be to perform orthology assignment based on the reciprocal smallest distance algorithm (RSD, [31]), which employs phylogenetics to distinguish between orthologs and paralogs. Though it would be technically possible to implement RSD in morFeus, this procedure is extremely time-consuming, as many sequences needed to be tested by RSD.

When should morFeus be used? morFeus is at its best, when a user searches the (co-)orthologs of a sequence with no close homologs in divergent species and therefore standard similarity search methods fail. If a sequence is a member of a larger protein family, for instance the kinase family, nuclear hormone receptors or Zinc fingers just to name a few, morFeus will not be the method of choice and phylogenetic approaches are better suited to identify orthologs. morFeus is however the method of choice when dealing with sequence orphans or sequences, where classical search methods only detect orthologs in closely related species.

## Conclusions

morFeus is the first web-based, fully automated method to detect remotely conserved orthologs of sequence orphans. We have realized this by 1) relaxing search parameters of BLAST to cover more sequence space of potential orthologs; 2) clustering resulting BLAST-alignments according to their similarity in order to identify conserved sequence patterns; 3) performing iterative reciprocal BLAST-searches to not only include orthology candidates that are picked up by more than one verified ortholog in previous rounds, but also to allow already confirmed orthologs, which fulfil the reciprocal best hit (RBH) relationship with the query to serve as RBH-recipients for further candidates; 4) and finally, by introducing a measure of orthology that is independent of the BLAST E-value and is based on the connectivity of a protein in its network of orthology. Our method is equally specific in the detection of well-conserved orthologs and more sensitive in finding remotely conserved orthologs than other web-based software suites available in the field to date.

## Availability and requirements

**Project Name**: morFeus

**Project Web-page**: http://bio.biochem.mpg.de/morfeus/; https://sourceforge.net/p/morfeus/.

**Operating System**: source code: Linux/Unix; web-server: platform-independent;

**Programming Language**: Python, PHP and Java

**License**: GNU GPL.

## Electronic supplementary material

Additional file 1: **morFeus.tar.gz contains the source code as submitted to sourceforge.net.** (ZIP 63 KB)

Additional file 2: **Additional Information provides a detailed description of the algorithms used in the following steps of morFeus: 1) distance-based clustering of alignments; 2) cluster cutting; 3) reciprocal BLAST candidate selection and orthology verification by RBH.** It furthermore contains information on the web-server implementation, the choice of E-value and database and the formulas used for calculating recall, precision, accuracy and F1-score. Additional information also contains the formulas of all functions tested for cluster cutting, Additional file 2: Figures S1-S3 plus figure legends, as well as Additional file 2: Tables S2, S4, S12 and S14. (PDF 13 MB)

Additional file 3: Table S1: Randomly chosen datasets from morFeus that were used for testing different functions (c.1 – c.22) for fitting clusters levels. **Table S3.** All hits identified in the initial BLAST search with *S. pombe* Apc13. Positively identified orthologs are color-coded in bright red, the false negative hit from *S. purpuratus* is highlighted in dark red, false positive hits are highlighted in dark green. **Table S5.** Recall, Precision, Accuracy and F1-score of morFeus, HomoloGene and Inparanoid in comparison to each other. **Table S6.** original data of the comparison of morFeus to HomoloGene. **Table S7.** original data of the comparison of morFeus to Inparanoid. **Table S8.** original data of the comparison of HomoloGene to Inparanoid. **Table S9.** original data of the comparison of Inparanoid to HomoloGene. **Table S10.** additional annotation of orthologs by morFeus for HomoloGene groups; TP = true positive ortholog identification by morFeus; FP = false positive ortholog identification by morFeus. **Table S11.** Original data from morFeus searches of remotely conserved COX assembly factors. The Precision was calculated for each morFeus search. **Table S13.** original data of the comparison of morFeus and Ortho-Profile on 184 remotely conserved, mitochondrial proteins from fission and budding yeast [22]. (XLSX 709 KB)
