# Supplementary material for: morFeus: a web-based program to detect remotely conserved orthologs using symmetrical best hits and orthology network scoring
Source: BMC Bioinformatics. 2014 Aug 6;15(1):263. doi: 10.1186/1471-2105-15-263 (PMC4137093; doi:10.1186/1471-2105-15-263)
Supplement: Supplementary file 2 — Additional file 2: Additional Information provides a detailed description of the algorithms used in the following steps of morFeus: 1) distance-based clustering of alignments; 2) cluster cutting; 3) reciprocal BLAST candidate selection and orthology verification by RBH. It furthermore contains information on the web-server implementation, the choice of E-value and database and the formulas used for calculating recall, precision, accuracy and F1-score. Additional information also contains the formulas of all functions tested for cluster cutting, Additional file 2: Figures S1-S3 plus figure legends, as well as Additional file 2: Tables S2, S4, S12 and S14. (PDF 13 MB) [file 12859_2014_6537_MOESM2_ESM.pdf]

Additional Material - Wagner, et al.

morFeus algorithms, implementation and performance testing

morFeus program and webserver. The morFeus back-end was implemented in Python, the web-interface was scripted in Python, PHP and Java. d3.js (http://d3js.org) is used to display the network of orthology.

morFeus works in four steps: 1) it performs relaxed BLAST-searches against a RefSeq-based protein database; 2) it clusters hits based on the similarity of their alignments to the query; 3) it performs iterative reciprocal BLAST searches, including hits that are found by more than one verified ortholog from previous rounds and allowing verified orthologs to function as reciprocal best hit for candidates from following iteration rounds; 4) it calculates a network score (based on Eigenvector Centrality as implemented in NetworkX) for each putative ortholog and therefore gives a measure of orthology independent of the BLAST E-value.

Choice of E-value and database: The setting of the BLAST E-value, as well as the chosen database will influence the results and run-time of morFeus. The higher the E-value, the more sequence space can be covered; however, a high E-value will also lengthen run time. A high E-value therefore only makes sense, if no or a limited number of homologs are found in a standard BLAST search by the query in distant organisms. The default E-value of a standard BLAST search at the NCBI is 10, covering a reasonable amount of sequence space. If a query does not find any homologs in more distant organisms (for instance a fungal protein does not have a hit in metazoans), we recommend an E-value of 100. If still no ortholog is found, the E-value can be raised up to 1000. In case the query is more conserved, we generally suggest using the standard E-value cut-off of 10 that is also used by NCBI-BLAST.

The chosen database will depend on the research question. In general, only strictly non-redundant databases can be used for morFeus searches. If queries have nearly identical sequences in the database (for instance only one or two amino acids differ due to sequencing variations), it will likely happen that the two nearly identical proteins compete for reciprocal best hits. Many true positives will therefore be excluded. It is for this reason that we chose the RefSeq databases as a well curated, truly unique protein databases as search space for morFeus. The query must be

contained within the chosen search database. It will result in no hits, if a bacterial sequence is for instance used to search in the Fungi/Metazoan search space. As a general guide, we recommend using smaller databases (subsets of RefSeq), as run-time will be shorter. Most orthology searches will also be restricted to either the bacterial or eukaryotic kingdom. It is therefore recommended to limit morFeus searches at least at this level.

Distance-based clustering of alignments: We have developed an appropriate scoring function to define the similarity between two alignments based on the position-specific comparison of two bit-strings, which represent the matches and mismatches of two query-subject alignments. First, the length of the alignment is set to the length n of the query, thus enabling the comparison between different BLAST alignments to the same query. In a next step, the alignment is transformed into a bit-string, representing all conserved positions as '1' and all non-conserved positions (or deletions) as '0'. The similarity $score_{\vec{a_l}\vec{a_k}}$ of two alignments $\vec{a_l} = (a_{l1}, \dots, a_{ln})$ and $\vec{a_k} = (a_{k1}, \dots, a_{kn})$ is then calculated as:

$$score_{\vec{a_l}\vec{a_k}} = \frac{\sum_{j=1}^{n} a_{lj} a_{kj} s_j}{\sqrt{(\vec{a_l}\vec{s})(\vec{a_k}\vec{s})} \times (\vec{d}\vec{s} + 10^{-20})} \qquad (1)$$

Here $\vec{d} = (d_1, \dots, d_n)$ represents the component-by-component difference between the two alignments, with $d_n = |a_{ln} - a_{kn}|$. The vector $\vec{s} = (s_1, \dots, s_n)$ refers to the substitution weight of the amino acid at a position j of the query in the OPTIMA substitution matrix [1]. We chose this matrix, as it is optimized to find remote orthologs and gives high scores for property-based conservation and substitutions. It outperformed in our hands substitution matrices like BLOSUM62 (data not shown). Finally, we treat conservation and property-based substitutions as equals, thereby assigning only the weight of the conserved amino acid of the query.

Given the $score_{\vec{a_l}\vec{a_k}}$, we can cluster the alignments on the basis of their similarity. Our clustering method is related to hierarchical clustering, so the two alignments with the highest score (i.e. which are closest to each other) are clustered first. The next step in hierarchical clustering is to determine a similarity score between the new cluster and the remaining alignments in the dataset and typically, a linkage method like complete, single or average linkage is chosen. However, we found any of these methods

inadequate to cluster alignments, as positional information of conservation is lost when only considering the $score_{\overrightarrow{a_l}\overrightarrow{a_k}}$. We therefore developed a modified average linkage approach. It is based on the conservation in terms of matches and mismatches at each position of a new cluster (c_g) and a single alignment or another cluster (c_h). $\vec{o} = (o_1, \dots, o_n)$ and $\vec{p} = (p_1, \dots, p_n)$ are two position-specific vectors we have introduced for this purpose. They can be interpreted as the percentage of matches and mismatches arising from the combination of two potential clusters at a certain position, given all sequences contained in the two clusters. For all positions $j = 1, \dots, n$ and all $l \in \{1, \dots, m\}$ with $a_l \in c_g$ and $k \in \{1, \dots, m\}$ with $a_k \in c_h$ the elements of the vectors $\vec{o}$ and $\vec{p}$ are calculated as:

$$o_j = s_j \frac{|\{a_{lj}|a_{lj}=1\}| \cdot |\{a_{kj}|a_{kj}=1\}|}{|\{a_l\}| \cdot |\{a_k\}|} \quad (2)$$

$$p_j = s_j \frac{|\{a_{lj}|a_{lj}=1\}| \cdot |\{a_{kj}|a_{kj}=0\}| + |\{a_{lj}|a_{lj}=0\}| \cdot |\{a_{kj}|a_{kj}=1\}|}{|\{a_l\}| \cdot |\{a_k\}|} \quad (3)$$

$\vec{o}$ and $\vec{p}$ are then employed to calculate the $score^*_{\overrightarrow{a_l}\overrightarrow{a_k}}$ for a pair of alignments $\overrightarrow{a_l} \in c_g$ and $\overrightarrow{a_k} \in c_h$.

$$score^*_{\overrightarrow{a_l}\overrightarrow{a_k}} = \frac{\sum_{j=1}^{n} a_{lj} a_{kj} o_j}{\sqrt{(\overrightarrow{a_l s})(\overrightarrow{a_k s})} \times (\vec{d}\vec{p} + 10^{-20})} \quad (4)$$

The final score for the similarity between c_g and c_h results from adding all $score^*_{\overrightarrow{a_l}\overrightarrow{a_k}}$ from all possible combinations of two alignments from c_g and c_h, respectively. The sum of scores* is therefore related to the number of possible combinations:

$$score_{c_g c_h} = \frac{\sum_{\{\vec{a}_l, \vec{a}_k | \vec{a}_l \in c_g, \vec{a}_k \in c_h\}} score^*_{\overrightarrow{a_l}\overrightarrow{a_k}}}{|\{\vec{a}_l \in c_g\}| \cdot |\{\vec{a}_k \in c_h\}|} \quad (5)$$

With our approach, each individual alignment contributes to the similarity score ($score_{c_g c_h}$) between two clusters. Each new alignment that is added to a cluster is therefore also compared against each alignment that is a member of the respective cluster. The output of the clustering of the BLAST hit alignments is a distance-based tree.

Cluster cutting: We cut the tree based on the specific distribution of distances ($distance_{c_g c_h} = \frac{1}{score_{c_g c_h}}$ (6)) within an individual distance tree. We first needed to determine a mathematical model that could describe the majority of our datasets. We therefore randomly chose 254 datasets and screened through various mathematical functions, including monomial and polynomial functions, as well as exponential functions (see Chapter '*Functions Tested for Cluster Cutting*' in this document, as well as Table S1 and Figure S1). Curve fitting was done using Gnuplot, which attempts to find a set of parameters that fits best the data to a defined mathematical model. As a starting point for fitting, we chose the default value of 1 for each parameter. To measure the quality of the fitting, we determined the sum of squared differences (SSD) of data points for each dataset and mathematical model and normalized it to the number of data points per dataset (NSSD). We observed that best fits could be achieved by polynomial functions (functions (***c.13)*** - (***c.22)***) as shown by the decreasing NSSD with increasing polynomial degree (Figure S1 a). Monomial functions with only one parameter (functions ***(c.3)*** - ***(c.12)***) showed a significantly higher NSSD, when compared to polynomial (functions ***(c.13)*** - ***(c.22)***), as well as the exponential function (function ***(c.1)***) and monomial functions with two parameters (function ***(c.2)***). We next analysed, how many datasets could be successfully fitted with the chosen functions (Figure S1 b). While functions ***(c.1.)*** - ***(c.12)*** (exponential and monomial functions) fitted nearly all tested datasets successfully, significantly less datasets could be fitted using the polynomial functions with increasing polynomial degree (functions ***(c.13)*** - ***(c.22)***). We reason that this is mostly due to insufficient data points for the number of parameters of higher degree polynomial functions. We therefore decided to use the exponential function to describe our datasets, since curve fitting worked in 97% of all cases and it displayed a satisfying fitting as evaluated by NSSD. Based on this function, the climbing rate can be used to determine cluster boundaries. The flatter the climbing rate, the higher the similarity. The flat climbing rate of an exponential curve therefore represents small clusters of high similarity. The larger the clusters, the lower the similarity – and hence the steeper the climbing rate will become. The point, where the climbing rate accelerates from a flat to a steep curve - in other words the region of climbing rate change (RcRc) – can be interpreted as the result of linking two more distantly related clusters

and consequently, the point (and distance score) we chose to separate the clusters of the hierarchical tree. For the purpose of determining RcRc of a specific hierarchical tree, we reduce the graph of the exponential function to the actual limits of the analysed tree. Point P_1, which refers to $level_1$ and point P_z, which refers to $level_z$ of a tree with z levels determine the two endpoints of the graph.

In order to find the point of steepest acceleration of the exponential curve, we introduce a linear function that defines a straight line between P_1 and P_z. RcRc is then determined by the intersection point P_{IP} of the exponential and tangent, which is derived from the linear function between P_1 and P_z.

From P_{IP} we can now calculate the Euclidian distance of any cluster of level r, in order to determine the cluster with the lowest distance to the intersection point, which is represented by P_{minED}.

We then define another linear function perpendicular to the exponential tangent through P_{IP} and determine where P_{minED} is located with respect to this perpendicular function. If P_{minED} is located in the area under the curve of the perpendicular function or exactly on that curve we consider the level represented by P_{minED} as cutting level, whereas if that criteria is not fulfilled, the next smaller level to P_{minED} is considered as level for cutting the tree.

In order to cover all possible sublevels of a given tree this procedure is performed individually for all alignments included within the tree, always starting from an individual alignment of $level_1$. In the case of mismatching decisions regarding these individual cutting levels of the tree, the level corresponding to the cluster that would include the maximal number of individual alignments is chosen for cutting the tree.

Finally in order to further reduce the number of reciprocal BLASTs to a reasonable amount we defined that if the sub-trees arising from cutting include more than 20 individual alignments, these clusters are then cut into further sub-clusters by applying the same procedure as described above.

Reciprocal BLAST candidate selection and orthology verification by RBH: To select candidates for reciprocal BLAST searches, we need to locate each alignment within the distance tree, as well as related members that are assigned to the respective clusters. Candidate selection is done iteratively and each validated ortholog is used to verify further hit candidates by the reciprocal best hit (RBH) rule, provided that it is

also the RBH of at least one other validated ortholog. At first, all highly homologous sequences (>80% identity) are selected for reciprocal BLASTs and validated against the query itself. From the second iteration onwards, all verified orthologs of this group are considered for the RBH-rule of newly selected candidates. Newly verified orthologs enlarge the group against which new candidates can be validated by the RBH-rule in the following iterations. We generally apply the RBH-rule non-stringently. In other words, protein pairs where only one of the two are found as the best hit in the respective species by the other protein, or which have an acceptable-acceptable relationship are not per se excluded as potential orthologs. However, if the E-value is below a certain threshold (E<10-5), the RBH-rule is applied strictly. For candidates with a higher E-value, hits that do not fulfil the RBH are considered as potential orthologs, if they fulfil the following criteria:

$$\text{E-value}_{cand} / \min(\text{E-value}[\text{species}_{cand}]) < 100 \qquad (6)$$

In addition to approval of new candidates, the group of orthologs can also exclude candidates, if they do not fulfil the RBH-rule, provided that more than 33% of the orthologs reject a potential candidate. Iterative testing for orthology continues until no new orthologs are found.

Calculation of Recall, Precision, Accuracy and F1-score: 190 HomoloGene groups were selected for testing the accuracy of morFeus (status of February 2012). In addition to the HomoloGene orthologs, the *Schizosaccharomyces pombe* (*S. pombe*, *Sp*) members of the families were annotated for orthologs by Inparanoid (stand-alone program, version 7, run with standard settings). morFeus was run using an E-value cut-off of 10 and resulting morFeus orthologs were cross-validated against the list of orthologs from HomoloGene and Inparanoid. Each protein family was manually verified and cleared of expired IDs (occurring for instance in HomoloGene) or unified identifiers for Mammalia/Vertebrae in the RefSeq database (status of April-July 2012). Based on the number of true positives (TP), false positives (FP), false negatives (FN) and true negatives (TN), we calculated Recall, Precision, Accuracy and F1-score for each family. For the overall scores, the average of all protein families were taken.

$$\text{Recall (Re)} = \frac{TP}{TP + FN} \quad (7)$$

$$\text{Precision (Pr)} = \frac{TP}{TP + FP} \quad (8)$$

$$\text{Accuracy (Ac)} = \frac{TP + TN}{TP + TN + FP + FN} \quad (9)$$

$$\text{F1-score} = 2 \times \frac{\text{Pr} \times \text{Re}}{\text{Pr} + \text{Re}} \quad (10)$$

morFeus searches were performed using net-BLAST [2]. For Inparanoid searches, we downloaded the genomes of *Saccharomyces cerevisiae*, *Schizosaccharomyces pombe*, *Neurospora crassa*, *Caenorhabditis elegans*, *Drosophila melanogaster*, *Danio rerio*, *Gallus gallus*, *Mus musculus*, *Rattus norvegicus* and *Homo sapiens* from the NCBI genome database and ran Inparanoid searches locally. The E-value cut-off was 10. Results were parsed using Perl and manually compared using Microsoft Excel®™. We manually resolved conflicts on accession number updates in the RefSeq database as compared to the ones present in HomoloGene groups. We also had to deal with the fusion of sequence entries in the RefSeq database – resulting in a frequent merging of many mammalian or vertebrate species into a single entry, if their protein sequences were identical. In those cases, we excluded the affected organisms from further analysis. All sequences in the morFeus hit-lists for Apc13 family were manually analysed for their orthologous relationship by reciprocal BLAST searches.

Comparison of morFeus against Ortho-Profile.

RefSeq sequence IDs were retrieved for the 598 proteins from *S. cerevisiae* described in [3] and first checked against HomoloGene. All those proteins that did not contain assigned orthologs in Metazoans were selected for further processing using morFeus. For those proteins that did not find a vertebrate/mammalian hit in the morFeus search with the yeast protein, a verified ortholog from another phylum (or class) was selected for further processing. If a hit from *S. pombe* was available, it was chosen for the iterative morFeus search.

Preparation of multiple sequence alignment and network graphics: Multiple sequence alignments were done manually based on the BLAST pair-wise sequence alignments and displayed using Adobe Illustrator^{TM}. Networks are displayed using Cytoscape [4]; prior to display, orthology networks were analysed using the plug-in NetworkAnalyzer (by Yassen Assenov (MPI for Informatics), Mario Albrecht (MPI for Informatics) and Mike Smoot (UCSD)).

Functions Tested for Cluster Cutting (see also Additional Table S1, Additional Figure S1)

functions

plot denomination	function formula
(c.1)	$f(x) = m * e^{x*k}$
(c.2)	$f(x) = m * x^{k}$
(c.3)	$f(x) = m * x^{1}$
(c.4)	$f(x) = m * x^{2}$
(c.5)	$f(x) = m * x^{3}$
(c.6)	$f(x) = m * x^{4}$
(c.7)	$f(x) = m * x^{5}$
(c.8)	$f(x) = m * x^{6}$
(c.9)	$f(x) = m * x^{7}$
(c.10)	$f(x) = m * x^{8}$
(c.11)	$f(x) = m * x^{9}$
(c.12)	$f(x) = m * x^{10}$
(c.13)	$f(x) = m * x^{1} + n$
(c.14)	$f(x) = m * x^{2} + n * x^{1} + o$
(c.15)	$f(x) = m * x^{3} + n * x^{2} + o * x^{1} + p$
(c.16)	$f(x) = m * x^{4} + n * x^{3} + o * x^{2} + p * x^{1} + q$
(c.17)	$f(x) = m * x^{5} + n * x^{4} + o * x^{3} + p * x^{2} + q * x^{1} + r$
(c.18)	$f(x) = m * x^{6} + n * x^{5} + o * x^{4} + p * x^{3} + q * x^{2} + r * x^{1} + s$
(c.19)	$f(x) = m * x^{7} + n * x^{6} + o * x^{5} + p * x^{4} + q * x^{3} + r * x^{2} + s * x^{1} + t$
(c.20)	$f(x) = m * x^{8} + n * x^{7} + o * x^{6} + p * x^{5} + q * x^{4} + r * x^{3} + s * x^{2} + t * x^{1} + u$
(c.21)	$f(x) = m * x^{9} + n * x^{8} + o * x^{7} + p * x^{6} + q * x^{5} + r * x^{4} + s * x^{3} + t * x^{2} + u * x^{1} + v$
(c.22)	$f(x) = m * x^{10} + n * x^{9} + o * x^{8} + p * x^{7} + q * x^{6} + r * x^{5} + s * x^{4} + t * x^{3} + u * x^{2} + v * x^{1} + w$

Additional Figure Legends

Figure S1: 254 randomly chosen datasets were fitted to selected mathematical functions using Gnuplot. For the monomial functions (grey) we chose fitting via one parameter (functions ***(c.3)*** - ***(c.12)***) or two parameters (function ***(c.2)***). Polynomal functions (green, functions ***(c.13)*** - ***(c.22)***) were fitted via n+1 parameters, where n refers to the degree of order of the polynomial function. In the case of the exponential function (pink, ***(c.1)***), the fitting was performed via two parameters (see Chapter '*Functions Tested for Cluster Cutting*' for details; see also Additional Table S1). **(a)** For each dataset and mathematical function, the sum of the squared differences (SSD) of data points was determined and normalized to the number of data points

(SSD/datapoint (NSSD)). SSD/datapoint (NSSD) was displayed for each function using the PRISM software. Bars extent from the smallest up to the largest value. Box boundaries represent the region from the 25th to the 75th percentiles. The line and the "+" in the middle of the box refer to the median and the mean respectively. **(b)** The number of successful curve fittings is displayed for each function.

Figure S2: Complete hit-list of the morFeus output for *S. pombe* Apc13 (NP_595754).

Figure S3: **(a)** Pair-wise BLAST alignments of the Apc13 family. *S. pombe* Apc13 was used as query, shown are the pair-wise alignments from more closely related species (*Schizosaccharomyces japonicus (S. japonicus)*, *Magnaporthe oryzae*) to the more distant alignments of zebrafish, the nematode *Loa loa* and mouse. **(b)** Pair-wise alignment of all mouse hits preceding and including the true Apc13 ortholog (NP_852059) with the query, *Sp* Ap13. **(c)** Pair-wise alignments of all false positive hits of the Apc13 family found by morFeus.
Highly conserved residues (present in the family) are highlighted in bright yellow, those that appear in most of them in dark yellow.

Additional Tables

Table S2: *S. pombe* Apc13 orthologs (70)

ID	Description	Network Score	E-value	Species
NP_595754.1	anaphase-promoting complex subunit Apc13	1.00	2.96885e-96	Schizosaccharomyces pombe 972h-
XP_003649350.1	hypothetical protein THITE_2169356	1.00	5.15061e-07	Thielavia terrestris NRRL 8126
XP_003050194.1	hypothetical protein NECHADRAFT_85069	0.99	2.33997e-08	Nectria haematococca mpVI 77-13-4
XP_003718297.1	Apc13 domain-containing protein	0.99	2.31648e-06	Magnaporthe oryzae 70-15
XP_002840575.1	hypothetical protein	0.98	5.02997e-06	Tuber melanosporum Mel28
XP_003661812.1	hypothetical protein MYCTH_36737, partial	0.97	0.000108498	Myceliophthora thermophila ATCC 42464
XP_386063.1	hypothetical protein FG05887.1	0.96	5.28697e-08	Fusarium graminearum PH-1
XP_001552519.1	hypothetical protein BC1G_08384	0.96	7.29176e-06	Botryotinia fuckeliana B05.10
XP_001595494.1	hypothetical protein SS1G_03583	0.96	7.50603e-06	Sclerotinia sclerotiorum 1980
XP_001225222.1	hypothetical protein CHGG_07566	0.94	0.000247443	Chaetomium globosum CBS 148.51
XP_001911394.1	hypothetical protein	0.92	0.0372369	Podospora anserina S mat+
XP_003855312.1	hypothetical protein MYCGRDRAFT_103444	0.92	0.107226	Zymoseptoria tritici IPO323
XP_002621260.1	conserved hypothetical protein	0.91	11.4232	Ajellomyces dermatitidis SLH14081
XP_001542346.1	predicted protein	0.91	28.9171	Ajellomyces capsulatus NAm1
XP_003010664.1	hypothetical protein ARB_03365	0.91	0.788237	Arthroderma benhamiae CBS 112371
XP_003170415.1	Apc13 domain-containing protein	0.91	1.42373	Arthroderma gypseum CBS 118893
XP_002567847.1	Pc21g08070	0.91	0.367234	Penicillium chrysogenum Wisconsin 54-1255
XP_003231001.1	Apc13 domain-containing protein	0.91	0.464304	Trichophyton rubrum CBS 118892
XP_002845195.1	Apc13 domain-containing protein	0.91	113.071	Arthroderma otae CBS 113480
XP_002795853.1	Apc13 domain-containing protein	0.91	92.4721	Paracoccidioides sp. 'lutzii' Pb01
XP_001274581.1	Apc13 domain protein	0.91	0.00491773	Aspergillus clavatus NRRL 1
XP_753351.1	Apc13 domain protein	0.91	0.00370773	Aspergillus fumigatus Af293
XP_001259388.1	Apc13 domain protein	0.91	0.00518092	Neosartorya fischeri NRRL 181
XP_664458.1	hypothetical protein AN6854.2	0.91	0.0542951	Aspergillus nidulans FGSC A4
XP_002381226.1	Apc13 domain protein	0.91	0.120585	Aspergillus flavus NRRL3357
XP_960666.2	hypothetical protein NCU08873	0.91	0.1123	Neurospora crassa OR74A
XP_001239650.1	hypothetical protein CIMG_09271	0.90	141.44	Coccidioides immitis RS
XP_003840023.1	hypothetical protein LEMA_P108090.1	0.90	48.8603	Leptosphaeria maculans JN3
XP_001800429.1	hypothetical protein SNOG_10147	0.90	4.88922	Phaeosphaeria nodorum SN15
XP_002145088.1	Apc13 domain protein	0.89	43.7407	Talaromyces marneffei ATCC 18224
XP_003002977.1	conserved hypothetical protein	0.89	1.54298	Verticillium alfalfae VaMs.102
XP_003346321.1	hypothetical protein SMAC_07970	0.89	0.453527	Sordaria macrospora k-hell
XP_001935825.1	conserved hypothetical protein	0.89	8.56047	Pyrenophora tritici-repentis Pt-1C-BFP
XP_002340295.1	Apc13 domain protein	0.88	106.897	Talaromyces stipitatus ATCC 10500
XP_003302441.1	hypothetical protein PTT_14246	0.87	251.979	Pyrenophora teres f. teres 0-1

XP_003188907.1	Apc13 domain protein	0.87	8.91418	Aspergillus niger CBS 513.88
XP_002175614.1	anaphase-promoting complex subunit Apc13	0.84	1.05055e-31	Schizosaccharomyces japonicus yFS275
XP_002617548.1	hypothetical protein CLUG_02992	0.38	2.92544	Clavispora lusitaniae ATCC 42720
XP_462101.2	DEHA2G12958p	0.36	0.0033186	Debaryomyces hansenii CBS767
XP_003708660.1	PREDICTED: anaphase-promoting complex subunit 13-like	0.29	170.171	Megachile rotundata
XP_001387686.1	predicted protein	0.27	101.509	Scheffersomyces stipitis CBS 6054
XP_004202164.1	Piso0_001647	0.27	2.66791	Millerozyma farinosa CBS 7064
XP_002549206.1	hypothetical protein CTRG_03503	0.27	6.20687	Candida tropicalis MYA-3404
XP_002590107.1	hypothetical protein BRAFLDRAFT_83385	0.25	773.636	Branchiostoma floridae
XP_002127489.1	PREDICTED: anaphase-promoting complex subunit 13-like	0.24	2.77027	Ciona intestinalis
XP_002420294.1	transcriptional activator, putative	0.24	3.41801	Candida dubliniensis CD36
XP_004069879.1	PREDICTED: anaphase-promoting complex subunit 13-like	0.23	76.5445	Oryzias latipes
XP_004202787.1	Piso0_001647	0.21	3.74494	Millerozyma farinosa CBS 7064
XP_003695866.1	PREDICTED: uncharacterized protein LOC100865957	0.19	129.004	Apis florea
NP_852059.1	anaphase-promoting complex subunit 13	0.18	829.486	Mus musculus
XP_001524637.1	predicted protein	0.18	178.612	Lodderomyces elongisporus NRRL YB-4239
XP_001483611.1	hypothetical protein PGUG_04340	0.15	0.137496	Meyerozyma guilliermondii ATCC 6260
NP_001167454.1	anaphase promoting complex subunit 13	0.15	907.948	Rattus norvegicus
XP_003967069.1	PREDICTED: anaphase-promoting complex subunit 13-like	0.15	40.0735	Takifugu rubripes
XP_003867211.1	Cta6 protein	0.15	15.1249	Candida orthopsilosis Co 90-125
XP_002666018.1	PREDICTED: anaphase-promoting complex subunit 13-like isoform X1	0.14	45.7128	Danio rerio
XP_003447091.1	PREDICTED: anaphase-promoting complex subunit 13-like	0.12	35.4838	Oreochromis niloticus
XP_002115489.1	hypothetical protein TRIADDRAFT_29505	0.12	446.239	Trichoplax adhaerens
NP_001140131.1	Anaphase-promoting complex subunit 13	0.12	38.8736	Salmo salar
NP_001187629.1	anaphase-promoting complex subunit 13	0.12	41.3102	Ictalurus punctatus
XP_005815366.1	PREDICTED: anaphase-promoting complex subunit 13-like	0.12	24.8711	Xiphophorus maculatus
XP_001897845.1	anaphase promoting complex subunit 13	0.11	467.461	Brugia malayi
XP_003137248.1	anaphase-promoting complex subunit 13	0.10	516.859	Loa loa
XP_715481.1	Cta6 protein extension	0.09	21.8588	Candida albicans SC5314
XP_002493722.1	hypothetical protein	0.06	643.437	Komagataella pastoris GS115
XP_314118.4	AGAP005216-PA	0.04	90.6356	Anopheles gambiae str. PEST
XP_001371885.1	PREDICTED: dopamine beta-hydroxylase-like	0.02	23.8234	Monodelphis domestica
XP_002915823.1	PREDICTED: alpha-1-acid glycoprotein-like	0.01	254.191	Ailuropoda melanoleuca
XP_004397008.1	PREDICTED: alpha-1-acid glycoprotein-like	0.01	284.892	Odobenus rosmarus divergens
XP_003211370.1	PREDICTED: dopamine beta-hydroxylase-like	0.00	709.742	Meleagris gallopavo

Table S4: *S. japonicus* Apc13 orthologs (98)

ID	Description	Network Score	E-value	Species
XP_001865218.1	conserved hypothetical protein	1.00	1.65296	Culex quinquefasciatus
XP_002175614.1	anaphase-promoting complex subunit Apc13	1.00	1.14085e-94	Schizosaccharomyces japonicus yFS275
XP_004664396.1	PREDICTED: anaphase-promoting complex subunit 13	0.99	207.442	Jaculus jaculus
XP_003708660.1	PREDICTED: anaphase-promoting complex subunit 13-like	0.98	29.4955	Megachile rotundata
XP_004069879.1	PREDICTED: anaphase-promoting complex subunit 13-like	0.97	24.1239	Oryzias latipes
NP_001165256.1	anaphase promoting complex subunit 13, gene 2	0.96	613.745	Xenopus laevis
XP_005863607.1	PREDICTED: anaphase-promoting complex subunit 13 isoform X1	0.96	182.025	Myotis brandtii
XP_002716344.1	PREDICTED: anaphase promoting complex subunit 13	0.96	185.723	Oryctolagus cuniculus
NP_001264411.1	anaphase-promoting complex subunit 13	0.96	253.625	Gallus gallus
XP_002590107.1	hypothetical protein BRAFLDRAFT_83385	0.96	81.7396	Branchiostoma floridae
NP_001127330.1	anaphase-promoting complex subunit 13	0.96	234.034	Pongo abelii
XP_001364784.1	PREDICTED: anaphase-promoting complex subunit 13-like	0.96	178.4	Monodelphis domestica
XP_005993753.1	PREDICTED: anaphase-promoting complex subunit 13-like	0.96	382.359	Latimeria chalumnae
XP_004698808.1	PREDICTED: anaphase-promoting complex subunit 13-like	0.95	189.496	Echinops telfairi
XP_005143125.1	PREDICTED: anaphase-promoting complex subunit 13	0.95	185.723	Melopsittacus undulatus
NP_001232060.2	anaphase-promoting complex subunit 13	0.95	197.272	Taeniopygia guttata
XP_006179771.1	PREDICTED: anaphase-promoting complex subunit 13	0.95	176.615	Camelus ferus
XP_002405168.1	anaphase-promoting complex subunit, putative	0.95	12.1342	Ixodes scapularis
XP_003218409.1	PREDICTED: anaphase-promoting complex subunit 13-like	0.95	512.274	Anolis carolinensis
XP_002127489.1	PREDICTED: anaphase-promoting complex subunit 13-like	0.95	6.32185	Ciona intestinalis
XP_005966651.1	PREDICTED: anaphase-promoting complex subunit 13 isoform X1	0.94	181.868	Pantholops hodgsonii
XP_004603018.1	PREDICTED: anaphase-promoting complex subunit 13	0.94	203.313	Sorex araneus
XP_003695866.1	PREDICTED: uncharacterized protein LOC100865957	0.94	3.63334	Apis florea
XP_006150907.1	PREDICTED: anaphase-promoting complex subunit 13 isoform X1	0.94	193.345	Tupaia chinensis
XP_005618721.1	PREDICTED: anaphase-promoting complex subunit 13-like	0.94	207.442	Canis lupus familiaris
XP_005303707.1	PREDICTED: anaphase-promoting complex subunit 13 isoform X1	0.94	92.6788	Chrysemys picta bellii
XP_001812217.1	PREDICTED: similar to conserved hypothetical protein	0.94	689.156	Tribolium castaneum
NP_852059.1	anaphase-promoting complex subunit 13	0.94	144.425	Mus musculus
NP_001017240.1	anaphase-promoting complex subunit 13	0.94	527.938	Xenopus (Silurana) tropicalis
NP_056206.1	anaphase-promoting complex subunit 13	0.93	183.865	Homo sapiens
XP_004477153.1	PREDICTED: anaphase-promoting complex subunit 13 isoform 1	0.93	161.329	Dasypus novemcinctus
XP_003501994.1	PREDICTED: anaphase-promoting complex subunit 13-like	0.93	89.0507	Cricetulus griseus
NP_001167454.1	anaphase promoting complex subunit 13	0.92	154.965	Rattus norvegicus
XP_003967069.1	PREDICTED: anaphase-promoting complex subunit 13-like	0.92	69.1941	Takifugu rubripes
NP_001140131.1	Anaphase-promoting complex subunit 13	0.91	272.105	Salmo salar
XP_005181107.1	PREDICTED: anaphase-promoting complex subunit 13-like	0.91	323.273	Musca domestica
NP_001187629.1	anaphase-promoting complex subunit 13	0.91	283.262	Ictalurus punctatus
XP_003447091.1	PREDICTED: anaphase-promoting complex subunit 13-like	0.91	94.6047	Oreochromis niloticus
XP_002666018.1	PREDICTED: anaphase-promoting complex subunit 13-like isoform X1	0.91	261.386	Danio rerio
XP_001184631.1	PREDICTED: anaphase-promoting complex subunit 13-like	0.91	150.668	Strongylocentrotus purpuratus
XP_005081548.1	PREDICTED: anaphase-promoting complex subunit 13-like	0.90	251.089	Mesocricetus auratus
XP_005815366.1	PREDICTED: anaphase-promoting complex subunit 13-like	0.90	55.9647	Xiphophorus maculatus
XP_002017598.1	GL17271	0.89	256.441	Drosophila persimilis

XP_002115489.1	hypothetical protein TRIADDRAFT_29505	0.89	956.473	Trichoplax adhaerens
XP_002005365.1	GI20436	0.89	399.326	Drosophila mojavensis
XP_002074911.1	GK23305	0.89	744.495	Drosophila willistoni
XP_004329239.1	PREDICTED: anaphase-promoting complex subunit 13-like	0.89	665.606	Tursiops truncatus
XP_001987638.1	GH19846	0.89	534.481	Drosophila grimshawi
XP_004588386.1	PREDICTED: anaphase-promoting complex subunit 13	0.89	57.1078	Ochotona princeps
XP_004414310.1	PREDICTED: anaphase-promoting complex subunit 13-like	0.87	993.84	Odobenus rosmarus divergens
XP_003718297.1	Apc13 domain-containing protein	0.84	2.0517e-05	Magnaporthe oryzae 70-15
XP_002840575.1	hypothetical protein	0.71	0.00444349	Tuber melanosporum Mel28
XP_003649350.1	hypothetical protein THITE_2169356	0.69	0.000172786	Thielavia terrestris NRRL 8126
XP_001552519.1	hypothetical protein BC1G_08384	0.61	0.000312652	Botryotinia fuckeliana B05.10
XP_001911394.1	hypothetical protein	0.56	2.7044	Podospora anserina S mat+
XP_001595494.1	hypothetical protein SS1G_03583	0.55	0.000407103	Sclerotinia sclerotiorum 1980
NP_595754.1	anaphase-promoting complex subunit Apc13	0.46	1.05055e-31	Schizosaccharomyces pombe 972h-
XP_003050194.1	hypothetical protein NECHADRAFT_85069	0.46	1.68587e-05	Nectria haematococca mpVI 77-13-4
XP_386063.1	hypothetical protein FG05887.1	0.37	0.000632672	Fusarium graminearum PH-1
XP_003661812.1	hypothetical protein MYCTH_36737, partial	0.36	0.0129903	Myceliophthora thermophila ATCC 42464
XP_001225222.1	hypothetical protein CHGG_07566	0.28	0.0367152	Chaetomium globosum CBS 148.51
XP_002429856.1	AN1-type zinc finger protein, putative	0.24	360.323	Pediculus humanus corporis
XP_003855312.1	hypothetical protein MYCGRDRAFT_103444	0.22	483.704	Zymoseptoria tritici IPO323
XP_960666.2	hypothetical protein NCU08873	0.22	0.25328	Neurospora crassa OR74A
XP_003170415.1	Apc13 domain-containing protein	0.21	14.3317	Arthroderma gypseum CBS 118893
XP_001800429.1	hypothetical protein SNOG_10147	0.21	83.651	Phaeosphaeria nodorum SN15
XP_001239650.1	hypothetical protein CIMG_09271	0.21	16.157	Coccidioides immitis RS
XP_003231001.1	Apc13 domain-containing protein	0.21	17.1472	Trichophyton rubrum CBS 118892
XP_002621260.1	conserved hypothetical protein	0.21	4.24975	Ajellomyces dermatitidis SLH14081
XP_003010664.1	hypothetical protein ARB_03365	0.21	13.496	Arthroderma benhamiae CBS 112371
XP_002381226.1	Apc13 domain protein	0.21	0.000147793	Aspergillus flavus NRRL3357
XP_001274581.1	Apc13 domain protein	0.21	0.000352707	Aspergillus clavatus NRRL 1
XP_002845195.1	Apc13 domain-containing protein	0.21	293.746	Arthroderma otae CBS 113480
XP_002795853.1	Apc13 domain-containing protein	0.21	109.513	Paracoccidioides sp. 'lutzii' Pb01
XP_753351.1	Apc13 domain protein	0.21	0.00159112	Aspergillus fumigatus Af293
XP_001259388.1	Apc13 domain protein	0.21	0.00136151	Neosartorya fischeri NRRL 181
XP_664458.1	hypothetical protein AN6854.2	0.21	0.00287887	Aspergillus nidulans FGSC A4
XP_002567847.1	Pc21g08070	0.21	0.023294	Penicillium chrysogenum Wisconsin 54-1255
XP_001542346.1	predicted protein	0.21	4.69794	Ajellomyces capsulatus NAm1
XP_002145088.1	Apc13 domain protein	0.20	1097.19	Talaromyces marneffei ATCC 18224
XP_003346321.1	hypothetical protein SMAC_07970	0.20	42.9838	Sordaria macrospora k-hell
XP_001935825.1	conserved hypothetical protein	0.20	2.05902	Pyrenophora tritici-repentis Pt-1C-BFP
XP_003840023.1	hypothetical protein LEMA_P108090.1	0.20	3.05581	Leptosphaeria maculans JN3
XP_002340295.1	Apc13 domain protein	0.20	550.449	Talaromyces stipitatus ATCC 10500
XP_003302441.1	hypothetical protein PTT_14246	0.19	12.1269	Pyrenophora teres f. teres 0-1
XP_003188907.1	Apc13 domain protein	0.19	53.5696	Aspergillus niger CBS 513.88
XP_314118.4	AGAP005216-PA	0.13	9.6824	Anopheles gambiae str. PEST
XP_462101.2	DEHA2G12958p	0.12	1.45298	Debaryomyces hansenii CBS767
XP_002617548.1	hypothetical protein CLUG_02992	0.12	35.6438	Clavispora lusitaniae ATCC 42720
XP_001387686.1	predicted protein	0.08	1019.97	Scheffersomyces stipitis CBS 6054
XP_002549206.1	hypothetical protein CTRG_03503	0.08	0.603037	Candida tropicalis MYA-3404
XP_002420294.1	transcriptional activator, putative	0.08	4.83727	Candida dubliniensis CD36
XP_004202164.1	Piso0_001647	0.07	0.412229	Millerozyma farinosa CBS 7064
XP_004202787.1	Piso0_001647	0.05	0.525803	Millerozyma farinosa CBS 7064
XP_001524637.1	predicted protein	0.05	451.36	Lodderomyces elongisporus NRRL YB-4239
XP_001483611.1	hypothetical protein PGUG_04340	0.03	0.0913639	Meyerozyma guilliermondii ATCC 6260
XP_003867211.1	Cta6 protein	0.03	6.49011	Candida orthopsilosis Co 90-125
XP_715481.1	Cta6 protein extension	0.00	35.1945	Candida albicans SC5314

Table S12: Algorithm comparison for 12 COX assembly factors from budding yeast

Gene Name	ID	KOG/COG	eggNog	HomoloGene	Berkley PHOG	FAT-CAT	Ortho-MCL
COX14	NP_013577	no hit	not found	no entry	fungi only	not found with FAT-CAT	fungi only
COX20	NP_010517	no hit	not found	fungi only	fungi only	FAT-CAT finds only arthropods	fungi only
COX23	NP_011984	found	found	fungi only	fungi only	found with FAT-CAT	found
COX24	NP_013305	no hit	not found	no entry	no entry	job not completed	fungi only
COA1	NP_012109	no hit	not found	fungi only	no entry	not found with FAT-CAT	fungi only
COA3	NP_076894	no hit	not found	fungi only	fungi only	not found with FAT-CAT	fungi only
MSS51	NP_013304	no hit	not found	fungi only	fungi only	not found with FAT-CAT	fungi only
PET100	NP_010364	no hit	not found	fungi only	not found	found with FAT-CAT	fungi only
PET117	NP_010979	no hit	not found	fungi only	not found	found with FAT-CAT	found
PET191	NP_012568	found invertebrates	found	fungi only	not found	not found with FAT-CAT	found
PET309	NP_013168	no hit	not found	fungi only	not found	not found with FAT-CAT	fungi only
YMR244C-A (COA6)	NP_013972	found	found	fungi only	not found	found with FAT-CAT	found

Table S14: Abbreviations and Accession Numbers for Figure S2 b.

**Apc13**	Sp	*Schizosaccharomyces pombe*	NP_595754
	Cag	*Candida glabrata*	XP_449727
	Ll	*Loa loa*	XP_003137248
	Mr	*Megachile rotundata*	XP_003708660
	Dr	*Danio rerio*	XP_002666018
	Mm	*Mus musculus*	NP_852059
	Os	*Oryza sativa*	NP_001060376

References in Additional Material (all included in main manuscript)

1. Kann M, Qian B, Goldstein RA: **Optimization of a new score function for the detection of remote homologs**. *Proteins* 2000, **41**(4):498-503.
2. Altschul SF, Madden TL, Schaffer AA, Zhang J, Zhang Z, Miller W, Lipman DJ: **Gapped BLAST and PSI-BLAST: a new generation of protein database search programs**. *Nucleic Acids Res* 1997, **25**(17):3389-3402.
3. Szklarczyk R, Wanschers BF, Cuypers TD, Esseling JJ, Riemersma M, van den Brand MA, Gloerich J, Lasonder E, van den Heuvel LP, Nijtmans LG *et al*: **Iterative orthology prediction uncovers new mitochondrial proteins and identifies C12orf62 as the human ortholog of COX14, a protein involved in the assembly of cytochrome c oxidase**. *Genome biology* 2012, **13**(2):R12.
4. Smoot ME, Ono K, Ruscheinski J, Wang PL, Ideker T: **Cytoscape 2.8: new features for data integration and network visualization**. *Bioinformatics* 2011, **27**(3):431-432.

a

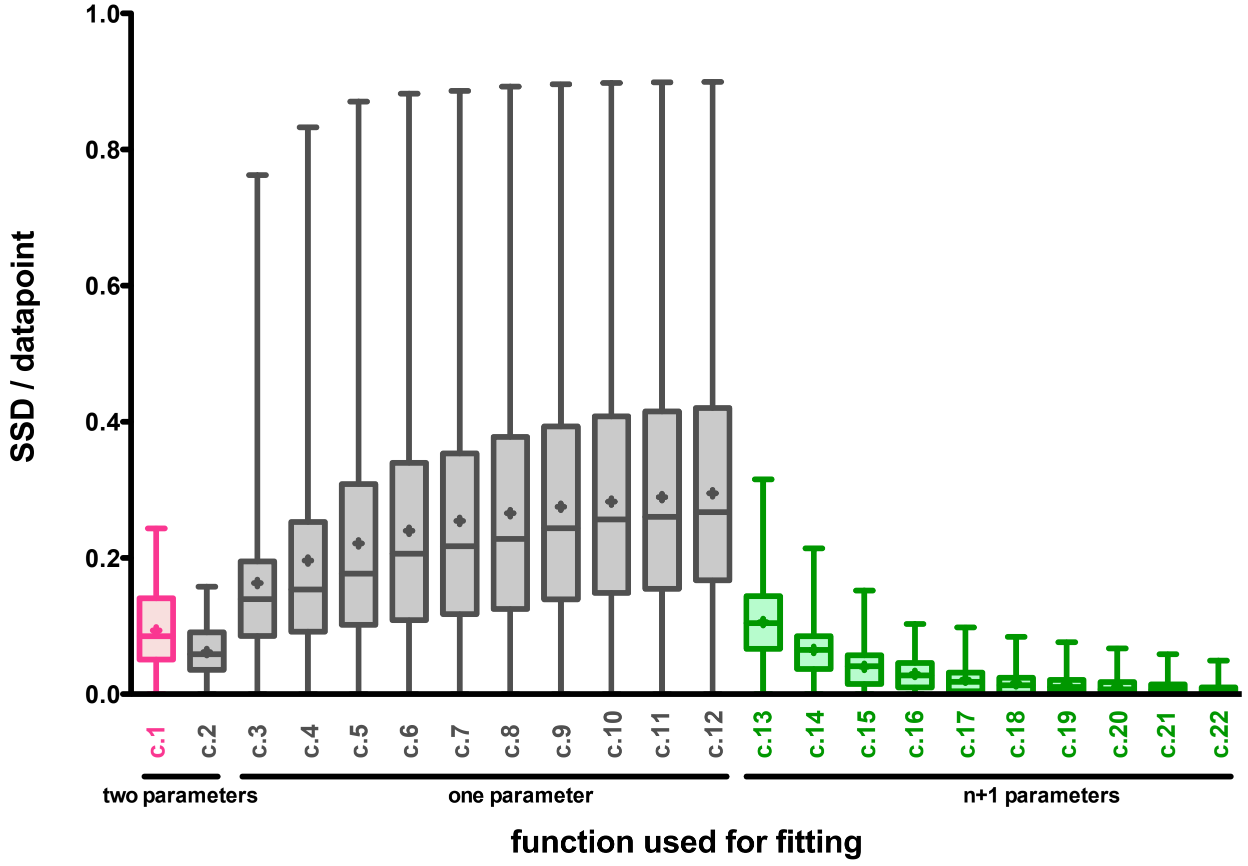

1.0
0.8
0.6
0.4
0.2
0.0
SSD / datapoint
c.1
c.2
c.3
c.4
c.5
c.6
c.7
c.8
c.9
c.10
c.11
c.12
c.13
c.14
c.15
c.16
c.17
c.18
c.19
c.20
c.21
c.22
two parameters
one parameter
n+1 parameters
function used for fitting

b

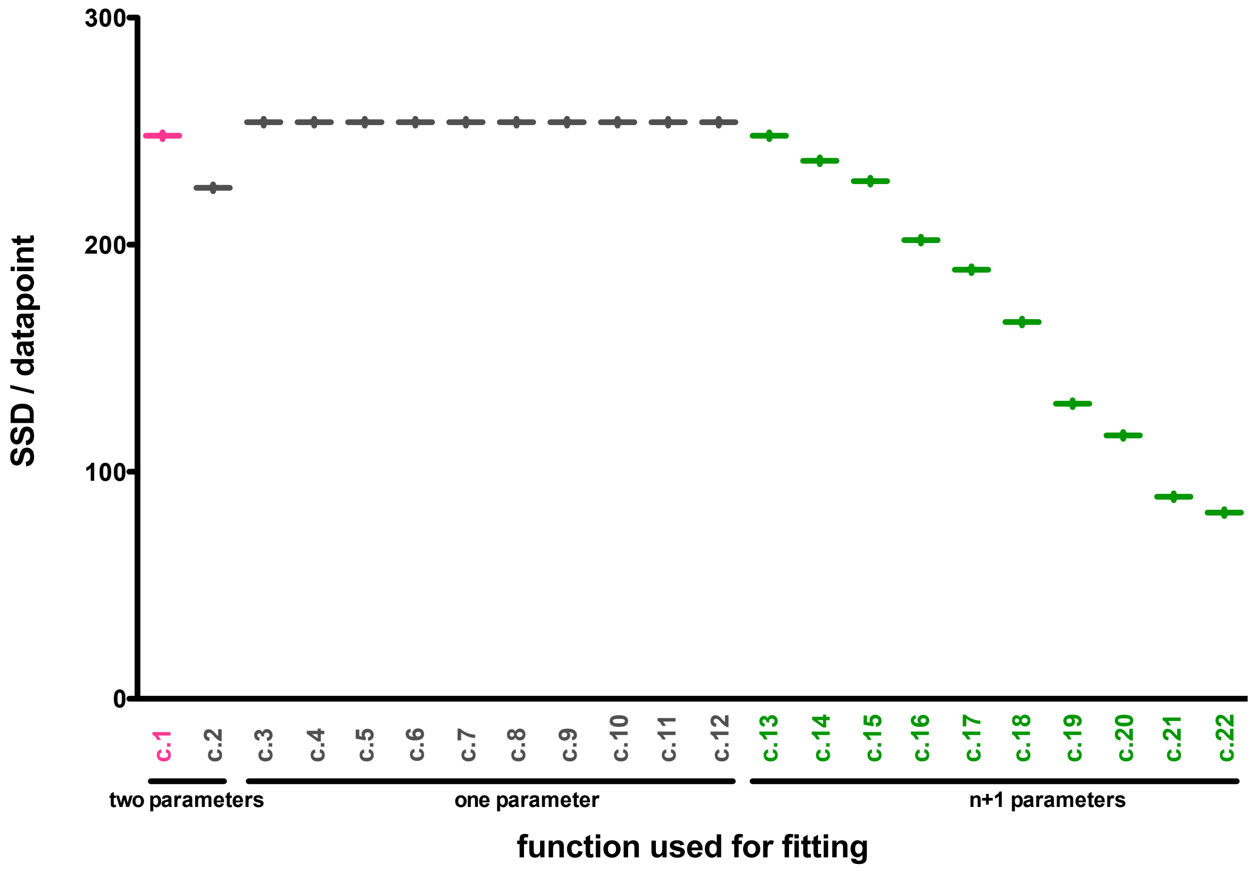

300
200
100
0
SSD / datapoint
c.1
c.2
c.3
c.4
c.5
c.6
c.7
c.8
c.9
c.10
c.11
c.12
c.13
c.14
c.15
c.16
c.17
c.18
c.19
c.20
c.21
c.22
two parameters
one parameter
n+1 parameters
function used for fitting

Figure S2

Submit | Process | Result | Network

Putative Orthologs for NP_595754.1

ID	Description	Network Score	Evalue	Species	Back BLAST	Alignment
NP_595754.1	anaphase-promoting complex subunit Apc13	1.00	2.96885e-96	Schizosaccharomyces pombe 972h-	BLAST output	Alignment
XP_003649350.1	hypothetical protein THITE_2169356	1.00	5.15061e-07	Thielavia terrestris NRRL 8126	BLAST output	Alignment
XP_003050194.1	hypothetical protein NECHADRAFT_85069	0.99	2.33997e-08	Nectria haematococca mpVI 77-13-4	BLAST output	Alignment
XP_003718297.1	Apc13 domain-containing protein	0.99	2.31648e-06	Magnaporthe oryzae 70-15	BLAST output	Alignment
XP_002840575.1	hypothetical protein	0.98	5.02997e-06	Tuber melanosporum Mel28	BLAST output	Alignment
XP_003661812.1	hypothetical protein MYCTH_36737, partial	0.97	0.000108498	Myceliophthora thermophila ATCC 42464	BLAST output	Alignment
XP_386063.1	hypothetical protein FG05887.1	0.96	5.28697e-08	Fusarium graminearum PH-1	BLAST output	Alignment
XP_001552519.1	hypothetical protein BC1G_08384	0.96	7.29176e-06	Botryotinia fuckeliana B05.10	BLAST output	Alignment
XP_001595494.1	hypothetical protein SS1G_03583	0.96	7.50603e-06	Sclerotinia sclerotiorum 1980	BLAST output	Alignment
XP_001225222.1	hypothetical protein CHGG_07566	0.94	0.000247443	Chaetomium globosum CBS 148.51	BLAST output	Alignment
XP_001911394.1	hypothetical protein	0.92	0.0372369	Podospora anserina S mat+	BLAST output	Alignment
XP_003855312.1	hypothetical protein MYCGRDRAFT_103444	0.92	0.107226	Zymoseptoria tritici IPO323	BLAST output	Alignment
XP_002621260.1	conserved hypothetical protein	0.91	11.4232	Ajellomyces dermatitidis SLH14081	BLAST output	Alignment
XP_001542346.1	predicted protein	0.91	28.9171	Ajellomyces capsulatus NAm1	BLAST output	Alignment
XP_003010664.1	hypothetical protein ARB_03365	0.91	0.788237	Arthroderma benhamiae CBS 112371	BLAST output	Alignment
XP_003170415.1	Apc13 domain-containing protein	0.91	1.42373	Arthroderma gypseum CBS 118893	BLAST output	Alignment
XP_002567847.1	Pc21g08070	0.91	0.367234	Penicillium chrysogenum Wisconsin 54-1255	BLAST output	Alignment
XP_003231001.1	Apc13 domain-containing protein	0.91	0.464304	Trichophyton rubrum CBS 118892	BLAST output	Alignment
XP_002845195.1	Apc13 domain-containing protein	0.91	113.071	Arthroderma otae CBS 113480	BLAST output	Alignment
XP_002795853.1	Apc13 domain-containing protein	0.91	92.4721	Paracoccidioides sp. 'lutzii' Pb01	BLAST output	Alignment
XP_001274581.1	Apc13 domain protein	0.91	0.00491773	Aspergillus clavatus NRRL 1	BLAST output	Alignment
XP_753351.1	Apc13 domain protein	0.91	0.00370773	Aspergillus fumigatus Af293	BLAST output	Alignment
XP_001259388.1	Apc13 domain protein	0.91	0.00518092	Neosartorya fischeri NRRL 181	BLAST output	Alignment
XP_664458.1	hypothetical protein AN6854.2	0.91	0.0542951	Aspergillus nidulans FGSC A4	BLAST output	Alignment
XP_002381226.1	Apc13 domain protein	0.91	0.120585	Aspergillus flavus NRRL3357	BLAST output	Alignment
XP_960666.2	hypothetical protein NCU08873	0.91	0.1123	Neurospora crassa OR74A	BLAST output	Alignment
XP_001239650.1	hypothetical protein CIMG_09271	0.90	141.44	Coccidioides immitis RS	BLAST output	Alignment
XP_003840023.1	hypothetical protein LEMA_P108090.1	0.90	48.8603	Leptosphaeria maculans JN3	BLAST output	Alignment
XP_001800429.1	hypothetical protein SNOG_10147	0.90	4.88922	Phaeosphaeria nodorum SN15	BLAST output	Alignment
XP_002145088.1	Apc13 domain protein	0.89	43.7407	Talaromyces marneffei ATCC 18224	BLAST output	Alignment
XP_003002977.1	conserved hypothetical protein	0.89	1.54298	Verticillium alfalfae VaMs.102	BLAST output	Alignment
XP_003346321.1	hypothetical protein SMAC_07970	0.89	0.453527	Sordaria macrospora k-hell	BLAST output	Alignment
XP_001935825.1	conserved hypothetical protein	0.89	8.56047	Pyrenophora tritici-repentis Pt-1C-BFP	BLAST output	Alignment
XP_002340295.1	Apc13 domain protein	0.88	106.897	Talaromyces stipitatus ATCC 10500	BLAST output	Alignment
XP_003302441.1	hypothetical protein PTT_14246	0.87	251.979	Pyrenophora teres f. teres 0-1	BLAST output	Alignment
XP_003188907.1	Apc13 domain protein	0.87	8.91418	Aspergillus niger CBS 513.88	BLAST output	Alignment
XP_002175614.1	anaphase-promoting complex subunit Apc13	0.84	1.05055e-31	Schizosaccharomyces japonicus yFS275	BLAST output	Alignment
XP_002617548.1	hypothetical protein CLUG_02992	0.38	2.92544	Clavispora lusitaniae ATCC 42720	BLAST output	Alignment
XP_462101.2	DEHA2G12958p	0.36	0.0033186	Debaryomyces hansenii CBS767	BLAST output	Alignment
XP_003708660.1	PREDICTED: anaphase-promoting complex subunit 13-like	0.29	170.171	Megachile rotundata	BLAST output	Alignment
XP_001387686.1	predicted protein	0.27	101.509	Scheffersomyces stipitis CBS 6054	BLAST output	Alignment
XP_004202164.1	Piso0_001647	0.27	2.66791	Millerozyma farinosa CBS 7064	BLAST output	Alignment
XP_002549206.1	hypothetical protein CTRG_03503	0.27	6.20687	Candida tropicalis MYA-3404	BLAST output	Alignment
XP_002590107.1	hypothetical protein BRAFLDRAFT_83385	0.25	773.636	Branchiostoma floridae	BLAST output	Alignment
XP_002127489.1	PREDICTED: anaphase-promoting complex subunit 13-like	0.24	2.77027	Ciona intestinalis	BLAST output	Alignment
XP_002420294.1	transcriptional activator, putative	0.24	3.41801	Candida dubliniensis CD36	BLAST output	Alignment
XP_004069879.1	PREDICTED: anaphase-promoting complex subunit 13-like	0.23	76.5445	Oryzias latipes	BLAST output	Alignment
XP_004202787.1	Piso0_001647	0.21	3.74494	Millerozyma farinosa CBS 7064	BLAST output	Alignment
XP_003695866.1	PREDICTED: uncharacterized protein LOC100865957	0.19	129.004	Apis florea	BLAST output	Alignment
NP_852059.1	anaphase-promoting complex subunit 13	0.18	829.486	Mus musculus	BLAST output	Alignment
XP_001524637.1	predicted protein	0.18	178.612	Lodderomyces elongisporus NRRL YB-4239	BLAST output	Alignment
XP_001483611.1	hypothetical protein PGUG_04340	0.15	0.137496	Meyerozyma guilliermondii ATCC 6260	BLAST output	Alignment
NP_001167454.1	anaphase promoting complex subunit 13	0.15	907.948	Rattus norvegicus	BLAST output	Alignment
XP_003967069.1	PREDICTED: anaphase-promoting complex subunit 13-like	0.15	40.0735	Takifugu rubripes	BLAST output	Alignment
XP_003867211.1	Cta6 protein	0.15	15.1249	Candida orthopsilosis Co 90-125	BLAST output	Alignment
XP_002666018.1	PREDICTED: anaphase-promoting complex subunit 13-like isoform X1	0.14	45.7128	Danio rerio	BLAST output	Alignment
XP_003447091.1	PREDICTED: anaphase-promoting complex subunit 13-like	0.12	35.4838	Oreochromis niloticus	BLAST output	Alignment
XP_002115489.1	hypothetical protein TRIADDRAFT_29505	0.12	446.239	Trichoplax adhaerens	BLAST output	Alignment
NP_001140131.1	Anaphase-promoting complex subunit 13	0.12	38.8736	Salmo salar	BLAST output	Alignment
NP_001187629.1	anaphase-promoting complex subunit 13	0.12	41.3102	Ictalurus punctatus	BLAST output	Alignment
XP_005815366.1	PREDICTED: anaphase-promoting complex subunit 13-like	0.12	24.8711	Xiphophorus maculatus	BLAST output	Alignment
XP_001897845.1	anaphase promoting complex subunit 13	0.11	467.461	Brugia malayi	BLAST output	Alignment
XP_003137248.1	anaphase promoting complex subunit 13	0.10	516.859	Loa loa	BLAST output	Alignment
XP_715481.1	Cta6 protein extension	0.09	21.8588	Candida albicans SC5314	BLAST output	Alignment
XP_002493722.1	hypothetical protein	0.06	643.437	Komagataella pastoris GS115	BLAST output	Alignment
XP_314118.4	AGAP005216-PA	0.04	90.6356	Anopheles gambiae str. PEST	BLAST output	Alignment
XP_001371885.1	PREDICTED: dopamine beta-hydroxylase-like	0.02	23.8234	Monodelphis domestica	BLAST output	Alignment
XP_002915823.1	PREDICTED: alpha-1-acid glycoprotein-like	0.01	254.191	Ailuropoda melanoleuca	BLAST output	Alignment
XP_004397008.1	PREDICTED: alpha-1-acid glycoprotein-like	0.01	284.892	Odobenus rosmarus divergens	BLAST output	Alignment
XP_003211370.1	PREDICTED: dopamine beta-hydroxylase-like	0.00	709.742	Meleagris gallopavo	BLAST output	Alignment

a

```
XP_002175614.1 anaphase-promoting complex subunit Apc13 [Schizosaccharomyces japonicus yFS275]
Sequence length of hit = 135
Score = 294, E = 1.05055e-31, Identities = 56/ 135 (41.5%), Positives = 84/ 135 (62.2%), Length = 135

Query: 1      MDSNYNYVHMNKPGVVLFASDWLKDRLPVDDVEVRVEHLPPVTEDEMTIQHSSANLILMKNKQLRHEPAWKDLELEDLVN   80
               MDS.Y+.+HM.+P..VL+A..WL+D+LPVDD++V.++.LPP+.+DE+.IQ.SS++LIL.+NKQ.+.+P.W.D+.LE.L+.
Sbjct: 1      MDSQYSQIHMQRPRTVLYAPTWLQDKLPVDDIDVGLQQLPPLPDDEVAIQQSSSHLILTRNKQQKTQPVWTDMGLETLIQ   80

Query: 81     AFAFIQGSSNAEGKNTIEDNFETDPFKSVKEAPMAPFLEANRRHQGEHASMRYFR     135
                .+...+.....E........F++......+..PM...LEANRRHQ.E.+++R.FR
Sbjct: 81     QYLDSEEIDKIEVYQHQYSYFKSKKNNETERLPMQFQLEANRRHQSETSNVRAFR     135


XP_003718297.1 Apc13 domain-containing protein [Magnaporthe oryzae 70-15]
Sequence length of hit = 128
Score = 117, E = 2.31648e-06, Identities = 30/ 82 (36.6%), Positives = 42/ 82 (51.2%), Length = 82

Query: 2      DSNYNYVHMNKPGVVLFASDWLKDRLPVDDVEVRVEHLPPVTEDEMTI---QHSSANLILMKNKQLRHEPAWKDLELEDL   81
               D..Y.+VHM.+........D+.KD+LP.DD+.V...H.P...EDE..+...QH++..+...K..Q...EPAW+DL.L.+L
Sbjct: 6      DGCYTHVHMRQARDADLFEDFCKDKLPADDIFVPPHHQPINPEDEDDVVPDQHAAFGIT--KATQKTKEPAWRDLGLSEL   85

Query: 82     VN      80
                +.
Sbjct: 86     MT      85


XP_002666018.1 PREDICTED: anaphase-promoting complex subunit 13-like isoform X1 [Danio rerio]
Sequence length of hit = 74
Score = 63, E = 45.7128, Identities = 21/ 72 (29.2%), Positives = 35/ 72 (48.6%), Length = 72

Query: 9      HMNKPGVVLFASD--WLKDRLPVDDVEVRVEHLPPVTEDEMTIQHSSANLILMKNKQLRHEPAWKDLELEDL   78
               .+.+.G.VL..+D..W.+DRLP.+DV.+.+..LP...+D......S......+K.++++....W.DL.L+.L
Sbjct: 4      EVQRDGRVLDLTDDAWREDRLPYEDVTIPLSELPEAEQDNGGSTES------VKEQEMK----WSDLALQSL   65


XP_003137248.1 anaphase promoting complex subunit 13 [Loa loa]
High-scoring segment pair (HSP) group
Score = 56, E = 516.859, Identities = 12/ 26 (46.2%), Positives = 18/ 26 (69.2%), Length = 26

Query: 21     DWLKDRLPVDDVEVRVEHLP-PVTED    45
               DW+..+LP.+.+E+..EHLP.P.TE+
Sbjct: 20     DWISHQLPKESIEIPREHLPDPETEN    45


NP_852059.1 anaphase-promoting complex subunit 13 [Mus musculus]
Sequence length of hit = 74
Score = 54, E = 829.486, Identities = 17/ 62 (27.4%), Positives = 29/ 62 (46.8%), Length = 62

Query: 17  LFASDWLKDRLPVDDVEVRVEHLPPVTEDEMTIQHSSANLILMKNKQLRHEPAWKDLELEDL 78
            L....W.+D+LP.+DV.+.+..LP...+D......S......+K.++++....W.DL.L+.L
Sbjct: 14  LIDDAWREDKLPYEDVAIPLSELPEPEQDNGGTTES------VKEQEMK----WTDLALQGL 65
```

b

```
NP_033010.2 receptor-type tyrosine-protein phosphatase mu precursor [Mus musculus]
Sequence length of hit = 1452
Score = 63, E = 195.886, Identities = 13/ 31 (41.9%), Positives = 18/ 31 (58.1%), Length = 31

Query: 19   ASDWLKDRLPVDDVEVRVEHLPPVTEDEMTI  49
             AS....DR.PVD....++.HL.P.TE.E+++
Sbjct: 323  ASGSWNDRQPVDSTSYKIGHLDPDTEYEISV  353
```

```
NP_032995.1 prostaglandin G/H synthase 1 precursor [Mus musculus]
Sequence length of hit = 602
=Score = 58, E = 714.643, Identities = 16/ 45 (35.6%), Positives = 27/ 45 (60%), Length = 45

Query: 13   PGVVLFASDWLKDRLPVDDVEVRVEHLPPVTEDEMTIQHSSANLI   57
             PG++LF++.WL++...V.D+.++.EH..P..+DE...Q.+...LI
Sbjct: 298  PGLMLFSTIWLREHNRVCDL-LKEEH--PTWDDEQLFQTTRLILI   339
```

```
NP_852059.1 anaphase-promoting complex subunit 13 [Mus musculus]
Sequence length of hit = 74
Score = 54, E = 829.486, Identities = 17/ 62 (27.4%), Positives = 29/ 62 (46.8%), Length = 62

Query: 17   LFASDWLKDRLPVDDVEVRVEHLPPVTEDEMTIQHSSANLILMKNKQLRHEPAWKDLELEDL 78
             L....W.+D+LP.+DV.+.+..LP...+D......S......+K.++++....W.DL.L+.L
Sbjct: 14   LIDDAWREDKLPYEDVAIPLSELPEPEQDNGGTTES------VKEQEMK----WTDLALQGL 65
```

C

```
XP_001371885.1 PREDICTED: dopamine beta-hydroxylase-like [Monodelphis domestica]
Length = 632
Score = 31.6 bits (70), Expect = 2.4e+01, Identities = 17, Positives = 31, Gaps = 6

Query:    5 YNY----VHMNKPGVVLFASDWLKDRLPVDDVEVRVEHLPPVTEDEMTIQHSSANLILMKNK   66
            YN+    +H NK   V F  +W +  LP   ++ R+E + P  +D   +  S++ L+ +  K
Sbjct:  571 YNFAPISMHCNKSSAVRFPGEWERQLLPT--IKTRLEEVTPQCQDTPRLSPSASTLVNIDGK  632
```

```
XP_002915823.1 PREDICTED: alpha-1-acid glycoprotein-like [Ailuropoda melanoleuca]
Length = 205
Score = 28.1 bits (61), Expect = 2.5e+02, Identities = 12, Positives = 20, Gaps = 0

Query:   40 PPVTEDEMTIQHSSANLILMKNKQLRHEPAWKDL   73
            P VTE++M + H +   I M+  ++ +  A KDL
Sbjct:  149 PEVTEEQMRVFHEAITCIGMQKSEISYTDAKKDL  182
```

```
XP_004397008.1 PREDICTED: alpha-1-acid glycoprotein-like [Odobenus rosmarus divergens]
Length = 206
Score = 28.1 bits (61), Expect = 2.8e+02, Identities = 12, Positives = 20, Gaps = 0

Query:   40 PPVTEDEMTIQHSSANLILMKNKQLRHEPAWKDL   73
            P VTE++M + H +   I M+  ++ +  A KDL
Sbjct:  150 PEVTEEQMRVFHEAIRCIGMQKSEISYTDAKKDL  183
```

```
XP_003211370.1 PREDICTED: dopamine beta-hydroxylase-like [Meleagris gallopavo]
Length = 616
Score = 26.9 bits (58), Expect = 7.1e+02, Identities = 14, Positives = 16, Gaps = 4

Query:    1 MDSNYNY----VHMNKPGVVLFASDWLKDRLP   32
            + S YN+    VH NK   V F  DW K  LP
Sbjct:  553 LKSLYNFAPISVHCNKSSAVRFPGDWEKQPLP  584
```
